# Supplementary material for: Selected Pharmaceuticals in Different Aquatic Compartments: Part II—Toxicity and Environmental Risk Assessment
Source: Molecules. 2020 Apr 14;25(8):1796. doi: 10.3390/molecules25081796 (PMC7221825; doi:10.3390/molecules25081796)
Supplement: Supplementary file 1 [file molecules-25-01796-s001.pdf]

Supporting information for

Selected pharmaceuticals in different aquatic compartments: Part II - toxicity and environmental risk assessment

André M.P.T. Pereira [a,*], Liliana J.G. Silva [a], Célia S.M. Laranjeiro [a], Celeste Lino [a], Angelina Pena [a]

[a] LAQV, REQUIMTE, Laboratory of Bromatology and Pharmacognosy, Faculty of Pharmacy, University of Coimbra, Polo III, Azinhaga de St[a] Comba, 3000-548 Coimbra, Portugal

***Corresponding author:**

LAQV, REQUIMTE, Group of Bromatology, Pharmacognosy and Analytical Sciences, Faculty of Pharmacy, University of Coimbra

Pólo das Ciências da Saúde, Azinhaga de Santa Comba, 3000-548 Coimbra, Portugal.

Tel: +351239488400

Fax : +351239827126

E-mail addresses:

amptpereira@gmail.com (A.M.P.T. Pereira), ljgsilva@hotmail.com (L.J.G. Silva), cmlino@ci.uc.pt (C. Lino), apena@ci.uc.pt (A. Pena)

Table S1. Ecotoxicological data on the selected pharmaceuticals.

Therapeutic group/ Pharmaceutical	Phylum(Class)	Specie (common name)	Acute toxicological endpoint	Acute ecotoxicity data	Chronic toxicological endpoint	Chronic ecotoxicity data	Other remarks	PNEC (ng L^{-1})	Reference
**Anxiolytics**									
**Alprazolam (ALP)**							**PNEC (ng L^{-1}) Algae – 892 Invertebrate – 3 590 Fish – 2 540**		
Algae		Green algae	EC50 (96 h)	0.892 mg L^{-1}			ECOSAR 2.0	892	
Invertebrate		Daphnid	LC50 (48 h)	3.59 mg L^{-1}			ECOSAR 2.0	3 590	
Fish			LC50 (96 h)	2.54 mg L^{-1}			ECOSAR 2.0	2 540	
**Lorazepam (LOR)**							**PNEC (ng L^{-1}) Algae - 6 070 Invertebrate - 39 400 Fish - 43 100**		
Algae		Green algae	EC50 (96 h)	6.07 mg L^{-1}			ECOSAR 2.0	6 070	
Invertebrate		Daphnid	LC50 (48 h)	39.4 mg L^{-1}			ECOSAR 2.0	39 400	
Fish			LC50 (96 h)	43.1 mg L^{-1}			ECOSAR 2.0	43 100	
**Zolpidem (ZOL)**							**PNEC (ng L^{-1}) Algae – 211 Invertebrate – 1 550 Fish – 248**		
Algae		Green algae	EC50 (96 h)	0.211 mg L^{-1}			ECOSAR 2.0	211	
Invertebrate		Daphnid	LC50 (48 h)	1.55 mg L^{-1}			ECOSAR 2.0	1 550	
Fish			LC50 (96 h)	0.248 mg L^{-1}			ECOSAR 2.0	248	

Therapeutic group/ Pharmaceutical	Phylum(Class)	Specie (common name)	Acute toxicological endpoint	Acute ecotoxicity data	Chronic toxicological endpoint	Chronic ecotoxicity data	Other remarks	PNEC (ng L^{-1})	Reference
**Antibiotics**									
**Azithromycin (AZI)**								**PNEC (ng L^{-1})** **Algae – 1.8 Invertebrate – 440 Fish – 84 000**	
Algae	Chlorophyta (Chlorophyceae)	Green algae (n.a)	EC50 (72h) (n.a)	0.0037 mg L^{-1}				3.7	[1]
		Blue-Green Algae (n.a)	EC50 (n.a)	0.0018 mg L^{-1}	NOEC (n.a)	0.00019 mg L^{-1}		1.8<br>19	[2]
Invertebrate	Arthropoda, Crustacea (Branchiopoda)	*Daphnia magna* (n.a)	EC50 (48h) (n.a)	120 mg L^{-1}				120 000	[1]
		Daphnia magna (planktonic crustacean)	EC50 (48h) (Immobilization)	100 mg L^{-1}				100 000	[3]
					NOEC (Chronic effects on reproduction, growth, and survival)	0.0044 mg L^{-1}		440	
Fish	Chordata (Actinopterygii)	*Oncorhynchus mykiss*	LC50 (96h) (n.a)	84 mg L^{-1}				84 000	[1]
					NOEC (Growth, survival, functioning, and reproduction)	4.6 mg L^{-1}		460 000	[3]
**Clarithromycin (CLA)**								**PNEC (ng L^{-1})** **Algae –** 2 **Invertebrate –** 8 160 **Fish –** 1 000 000	
Bacteria	Proteobacteria (Gammaproteobacteria)	*Vibrio fischeri*	EC50 (30 min) (luminescence)	100 mg L^{-1}				100 000	[4]
Algae	Chlorophyta (Chlorophyceae)	*Raphidocelis subcapitata (Selenastrum capricornutum,*	EC50 (72 h) (growth inhibition)<br>LOEC (72 h) (growth inhibition)	46 µg L^{-1}<br>40 µg L^{-1}				46<br>800	[5]

Therapeutic group/ Pharmaceutical	Phylum(Class)	Specie (common name)	Acute toxicological endpoint	Acute ecotoxicity data	Chronic toxicological endpoint	Chronic ecotoxicity data	Other remarks	PNEC (ng L^{-1})	Reference
		Pseudokirchneriella subcapitata)	EC50 (72 h) (growth inhibition)	0.0020 mg L^{-1}				2	[4]
Invertebrate	Rotifera (Monogononta)	*Brachionus calyciflorus*			LC50 (48 h) (growth inhibition)	12.21 mg L^{-1}		122 100	[4]
			LC50 (24 h) (mortality)	35.46 mg L^{-1}				35 460	
	Arthropoda, Crustacea (Branchiopoda)	*Ceriodaphnia dubia*	EC50 (24 h) (growth inhibition)	8.16 mg L^{-1}				8 160	[4]
			EC50 (48 h) (mobility)	18.66 mg L^{-1}				18 660	
		Thamnocephalus platyurus	LC50 (24 h) (mortality)	33.64 mg L^{-1}				33 640	[4]
		Daphnia magna	EC50 (24 h) (immobilisation test)	25.72 mg L^{-1}				25 720	[4]
Fish	Chordata (Actinopterygii)	*Danio rerio* (zebrafish)	LC50 (96 h) (mortality)	1 000 mg L^{-1}				1 000 000	[4]
**Ciprofloxacin (CIP)**								**PNEC (ng L^{-1})** **Algae –** 5 **Invertebrate –** 10 000 **Fish –** 1 000 000	
Bacteria		Sludge bacteria (n.a)	EC50 (6 h) (growth)	0.61 mg L^{-1}				610	[6]
	Proteobacteria (Gammaproteobacteria)	*Vibrio fischeri*	EC50 (30 min) (luminescence)	11.5 mg L^{-1}				11 500	[7]
Algae	Cyanobacteria (Nostocales)	*Anabaena flos-aquae*	NOEC (3 d) (yield)<br>EC50 (3 d) (biomass and growth rate)	5.65 µg L^{-1}<br>10.2 µg L^{-1}				113<br>10.2	[8]
	Cyanobacteria (Cyanophyceae)	*Microcystis aeruginosa*	EC50 (72 h) (growth inhibition)	0.005 mg L^{-1}				5	[6]
					EC50 (5 d) (growth)	17 µg L^{-1}		170	[9]

Therapeutic group/ Pharmaceutical	Phylum(Class)	Specie (common name)	Acute toxicological endpoint	Acute ecotoxicity data	Chronic toxicological endpoint	Chronic ecotoxicity data	Other remarks	PNEC (ng L^{-1})	Reference
	Chlorophyta (Chlorophyceae)	*Raphidocelis subcapitata (Selenastrum capricornutum, Pseudokirchneriella subcapitata)*	EC50 (72 h) (growth inhibition)	6.7 µg L^{-1}				6.7	[5]
			LOEC (72 h) (growth inhibition)	5 µg L^{-1}				100	
			EC50 (72 h) (growth inhibition)	2.97 mg L^{-1}				2 970	[6]
			EC50 (96 h) (Growth inhibition (cell density))	4.83 mg L^{-1}				4 830	[7]
			NOEC (96 h) (Growth inhibition (cell density))	1.09 mg L^{-1}				21 800	
			LOEC (96 h) (growth inhibition)	2.19 mg L^{-1}				43 800	
			EC50 (3 d) (growth)	18 700 µg L^{-1}				18 700	[9]
	Chlorophyta (Trebouxiophyceae)	*Chlorella vulgaris*	EC50 (96 h) (reduction in growth)	20.6 mg L^{-1}				20 600	[10]
	Tracheophyta (Liliopsida)	*Lemna gibba* (duckweed)			LOEC (7 d) (static tests)	300 µg L^{-1}		30 000	[11]
					EC50 (7 d) (static tests)	697 µg L^{-1}		6 970	
		Lemna minor			NOEC (7 d) (inhibition of yield)	10.0 µg L^{-1}		1 000	[8]
					EC50 (7 d) (growth)	62.5 µg L^{-1}		625	
					EC50 (7 d) (growth)	203 µg L^{-1}		2 030	[9]
					EC50 (7 d) (growth)	3.75 mg L^{-1}		37 500	[7]
Invertebrate		*Daphnia magna*	NOEC (48 h)	60 mg L^{-1}				1 200 000	[6]

Therapeutic group/ Pharmaceutical	Phylum(Class)	Specie (common name)	Acute toxicological endpoint	Acute ecotoxicity data	Chronic toxicological endpoint	Chronic ecotoxicity data	Other remarks	PNEC (ng L^{-1})	Reference
			(immobilization and reproduction)						
			EC50 (48 h) (immobilisation)	65.3 mg L^{-1}				65 300	[7]
					EC50 (21 d) (fecundity)	12.8 mg L^{-1}		128 000	[7]
					NOEC (21 d) (fecundity)	5.19 mg L^{-1}		519 000	
					LOEC (21 d) (fecundity of females)	8.82 mg L^{-1}		882 000	
	Arthropoda, Crustacea (Branchiopoda)				NOEC (21 d) (age at first reproduction)	15 mg L^{-1}		1 500 000	[7]
					LOEC (21 d) (age at first reproduction)	25.5 mg L^{-1}		2 550 000	
					NOEC (21 d) (number of broods per female)	8.82 mg L^{-1}		882 000	[7]
					LOEC (21 d) (number of broods per female)	15 mg L^{-1}		1 500 000	
					NOEC (21 d) (size of neonates from the first brood)	1.8 mg L^{-1}		180 000	[7]
					LOEC (21 d)	3.05 mg L^{-1}		305 000	

Therapeutic group/ Pharmaceutical	Phylum(Class)	Specie (common name)	Acute toxicological endpoint	Acute ecotoxicity data	Chronic toxicological endpoint	Chronic ecotoxicity data	Other remarks	PNEC (ng L^{-1})	Reference
					(size of neonates from the first brood)				
					NOEC (21 d) (somatic growth rate)	8.82 mg L^{-1}		882 000	[7]
					LOEC (21 d) (somatic growth rate)	15 mg L^{-1}		1 500 000	
					NOEC (21 d) (intrinsic rate of population increase)	8.82 mg L^{-1}		882 000	[7]
					LOEC (21 d) (intrinsic rate of population increase)	15 mg L^{-1}		1 500 000	
			EC50 (48h) (n.a)	10 mg L^{-1}				10 000	[1]
Fish	Chordata (Actinopterygii)	*Danio rerio* (zebrafish)	NOEC (72 h) (mortality)	100 mg L^{-1}				2 000 000	[6]
		Brachydanio rerio (zebrafish)	LC50 (48h) (n.a)	1 000 mg L^{-1}				1 000 000	[1]
**Erythromycin (ERY)**								**PNEC (ng L^{-1})** **Algae –** 20 **Invertebrate –** 220 **Fish –** 1 000 000	
Bacteria	Proteobacteria (Gammaproteobacteria)	*Vibrio fischeri*	EC50 (30 min) (luminescence)	100 mg L^{-1}				100 000	[4]
Algae	Chlorophyta (Chlorophyceae)	*Raphidocelis subcapitata (Selenastrum capricornutum, Pseudokirchneriella subcapitata)*	EC 50 (72 h) (growth inhibition)	0.020 mg L^{-1}				20	[4]
			EC50 (72h) (growth)	38 µg L^{-1}				38	[12]

Therapeutic group/ Pharmaceutical	Phylum(Class)	Specie (common name)	Acute toxicological endpoint	Acute ecotoxicity data	Chronic toxicological endpoint	Chronic ecotoxicity data	Other remarks	PNEC (ng L^{-1})	Reference
		Dunaliella tertiolecta	EC 50 (96h) (growth)	5 750 µg L^{-1}				5 750	[12]
Invertebrate	Rotifera (Monogononta)	*Brachionus calyciflorus*			LC50 (48 h) (growth inhibition)	0.94 mg L^{-1}		9 400	[4]
			LC50 (24 h) (mortality)	27.53 mg L^{-1}				27 530	
	Arthropoda, Crustacea (Branchiopoda))	*Thamnocephalus platyurus*	LC50 (24 h) (mortality)	17.68 mg L^{-1}				17 680	[4]
		Daphnia magna	EC50 (24 h) (immobilisation test)	22.45 mg L^{-1}				22 450	[4]
		Ceriodaphnia dubia	EC50 (48 h) (mobility)	10.23 mg L^{-1}				10 230	[4]
			EC50 (24 h) (growth inhibition)	0.22 mg L^{-1}				220	
Fish	Chordata (Actinopterygii)	*Danio rerio* (zebrafish)	LC50 (96 h) (mortality)	1 000 mg L^{-1}				1 000 000	[4]
**Lipid regulators**									
**Bezafibrate (BEZ)**							**PNEC (ng L^{-1})** **Algae –** 4 870 **Invertebrate –** 1 300 **Fish –** 17 600		
Algae		Green algae	EC50 (96 h)	4.87 mg L^{-1}			ECOSAR 2.0	4 870	
Invertebrate	Cnidaria (Hydrozoa)	*Hydra attenuata*	LC50 (96 h) (morphology)	70.71 mg L^{-1}				70 710	[13]
			EC50 (96 h) (morphology)	25.85 mg L^{-1}				25 850	
			LOEC (96 h) (morphology)	1 mg L^{-1}				20 000	
			NOEC (96 h) (morphology)	0.1 mg L^{-1}				2 000	

Therapeutic group/ Pharmaceutical	Phylum(Class)	Specie (common name)	Acute toxicological endpoint	Acute ecotoxicity data	Chronic toxicological endpoint	Chronic ecotoxicity data	Other remarks	PNEC (ng L^{-1})	Reference
			EC50 (96 h) (feeding)	8.59 mg L^{-1}				8 590	
	Rotifera (Monogononta)	*Brachionus calyciflorus*	LC50 (24 h) (mortality)	60.91 mg L^{-1}				60 910	[14]
					EC50 (48 h) (population growth inhibition)	0.44 mg L^{-1}	4 400		
					NOEC (48 h) (population growth inhibition)	0.156 mg L^{-1}	15 600	[14]	
					LOEC (48 h) (population growth inhibition)	0.3125 mg L^{-1}	31 250		
	Arthropoda, Crustacea (Branchiopoda)	*Thamnocephalus platyurus*	LC50 (24 h) (mortality)	39.69 mg L^{-1}				39 690	[14]
		Daphnia magna	EC50 (48 h) (immobilisation test)	100.08 mg L^{-1}				100 080	[14]
			EC50 (48 h) (mortality)	30.3 mg L^{-1}				30 300	[15]
		Ceriodaphnia dubia	EC50 (8 h) (immobilisation test)	75.79 mg L^{-1}				75 790	[14]
					EC50 (7 d) (population growth inhibition)	0.13 mg L^{-1}		1 300	
					NOEC (7 d) (population growth inhibition)	0.023 mg L^{-1}		2 300	

Therapeutic group/ Pharmaceutical	Phylum(Class)	Specie (common name)	Acute toxicological endpoint	Acute ecotoxicity data	Chronic toxicological endpoint	Chronic ecotoxicity data	Other remarks	PNEC (ng L^{-1})	Reference
					LOEC (7 d) (population growth inhibition)	0.047 mg L^{-1}		4 700	
Fish			LC50 (96 h)	17.6 mg L^{-1}			ECOSAR 2.0	17 600	
**Gemfibrozil (GEM)**								**PNEC (ng L^{-1})** **Algae –** 15 190 **Invertebrate –** 1 180 **Fish –** 150	
Bacteria	Proteobacteria (Gammaproteobacteria)	*Vibrio fischeri*	EC50 (30 min) (inhibition of luminescence)	85.74 mg L^{-1}				85 740	[14]
			EC50 (24 h)<br>EC50 (48 h) (inhibition of luminescence)	64.6 mg L^{-1}<br>45.1 mg L^{-1}				64 600<br>45 100	[16]
			EC50 (inhibition of luminescence)	18 800 µg L^{-1}<br>31 500 µg L^{-1}			Two test were assayed ToxAlert 100 Microtox	18 800<br>31 500	[17]
Algae	Chlorophyta (Chlorophyceae)	*Raphidocelis subcapitata (Selenastrum capricornutum, Pseudokirchneriella subcapitata)*	EC50 (72 h) (growth inhibition)<br>NOEC (72 h) (growth inhibition)<br>LOEC (72 h) (growth inhibition)	15.19 mg L^{-1}<br>3.125 mg L^{-1}<br>6.25 mg L^{-1}				15 190<br>62 500<br>125 000	[14]
	Chlorophyta (Trebouxiophyceae)	*Chlorella vulgaris*	EC50 (24 h) (growth inhibition test)	195 mg L^{-1}				195 000	[16]

Therapeutic group/ Pharmaceutical	Phylum(Class)	Specie (common name)	Acute toxicological endpoint	Acute ecotoxicity data	Chronic toxicological endpoint	Chronic ecotoxicity data	Other remarks	PNEC (ng L^{-1})	Reference
			EC50 (48 h) (growth inhibition test)	161 mg L^{-1}				161 000	
			EC50 (72 h) (growth inhibition test)	150 mg L^{-1}				150 000	
Invertebrate	Cnidaria (Hydrozoa)	*Hydra attenuata*	LC50 (96 h) (morphology)EC50 (96 h) (morphology)	22.36 mg L^{-1}<br>1.18 mg L^{-1}				22 360<br>1 180	[13]
			LOEC (96 h) (morphology)	1 mg L^{-1}				20 000	
			NOEC (96 h) (morphology)	0.1 mg L^{-1}				2 000	
			EC50 (96 h) (feeding response)	1.76 mg L^{-1}				1 760	
	Rotifera (Monogononta)	*Brachionus calyciflorus*	LC50 (24 h) (mortality)	77.30 mg L^{-1}				77 300	[14]
					EC50 (48 h) (population growth inhibition)	0.44 mg L^{-1}		4 400	
					NOEC (48 h) (population growth inhibition)	0.156 mg L^{-1}		15 600	
					LOEC (48 h) (population growth inhibition)	0.312 mg L^{-1}		31 200	
	Arthropoda, Crustacea (Branchiopoda)	*Thamnocephalus platyurus*	LC50 (24 h) (mortality)	161.05 mg L^{-1}				161 050	[14]
		Ceriodaphnia dubia			EC50 (7 d) (population	0.53 mg L^{-1}		5 300	[14]

Therapeutic group/ Pharmaceutical	Phylum(Class)	Specie (common name)	Acute toxicological endpoint	Acute ecotoxicity data	Chronic toxicological endpoint	Chronic ecotoxicity data	Other remarks	PNEC (ng L^{-1})	Reference
					growth inhibition)				
					NOEC (7 d) (population growth inhibition)	0.078 mg L^{-1}		7 800	
					LOEC (7 d) (population growth inhibition)	0.156 mg L^{-1}		15 600	
		Daphnia magna	EC50 (48 h) (acute immobilisation test)	74.30 mg L^{-1}				74 300	[14]
			EC50 (48 h) (mortality)	10.4 mg L^{-1}				10 400	[15]
			EC50 (24 h) (acute immobilisation test)	57.1 mg L^{-1}				57 100	[16]
			EC50 (48 h) (acute immobilisation test)	42.6 mg L^{-1}				42 600	
			EC50 (72 h) (acute immobilisation test)	30.0 mg L^{-1}				30 000	
Fish	Chordata (Actinopterygii)	*Carassius auratus*			LOEC (21 d) (induce oxidative stress in goldfish liver)	1.5 µg L^{-1}	(LOEC was not calculated in the paper)	150	[18]
	Chordata (Actinopterygii)	*Danio rerio* (zebrafish)	LOEC (5 d) (embryonic malabsorption syndrome)	5 mg L^{-1}			(LOEC was not calculated in the paper)	100 000	[19]

Therapeutic group/ Pharmaceutical	Phylum(Class)	Specie (common name)	Acute toxicological endpoint	Acute ecotoxicity data	Chronic toxicological endpoint	Chronic ecotoxicity data	Other remarks	PNEC (ng L^{-1})	Reference
**Simvastatin (SIM)**									**PNEC (ng L^{-1})** **Algae –** 22 800 **Invertebrate –** 3.2 **Fish –** 765
Algae	Chlorophyta (Chlorophyceae)	*Dunaliella tertiolecta*	EC50 (96 h) (growth inhibition)	22 800 µg L^{-1}				22 800	[20]
Invertebrate	Arthropoda, Crustacea (Malacostraca)	*Palaemonetes pugio* (Grass shrimp)	LC50 (96 h) (larval Toxicity Test)	1.18 mg L^{-1}				1 180	[21]
			NOEC (96 h) (adult toxicity test)	0.625 mg L^{-1}				12 500	
			LOEC (96 h) (larval Toxicity Test)	1.25 mg L^{-1}				25 000	
			LC50 (96 h) (adult toxicity test)	>10 mg L^{-1}				>10 000	
			NOEC (96 h) (adult toxicity test)	5.00 mg L^{-1}				100 000	
			LOEC (96 h) (adult toxicity test)	10.0 mg L^{-1}				200 000	
	Arthropoda ,Crustacea (Maxillopoda)	*Nitocra spinipes*	LC50 (96 h) (growth rate)	810 µg L^{-1}			Not calculated in the paper. RNA content and body length increased significantly at 0.16 µg L^{-1}	810	[22]
			LOEC (96 h) (growth rate)	0.16 µg L^{-1}				3.2	
Fish			LC50 (96 h)	0.765 mg L^{-1}			ECOSAR 2.0	765	

Therapeutic group/ Pharmaceutical	Phylum(Class)	Specie (common name)	Acute toxicological endpoint	Acute ecotoxicity data	Chronic toxicological endpoint	Chronic ecotoxicity data	Other remarks	PNEC (ng L^{-1})	Reference
**Antiepiletic**									
**Carbamazepine (CAR)**									**PNEC (ng L^{-1})** **Algae –** 31.6 **Invertebrate –** 0.2 **Fish –** 20 000
Algae	Cyanobacteria (Cyanophyceae)	*Synechococcus leopoliensis*	NOEC (96 h) (growth)	17.5 µg L^{-1}				350	[23]
			EC50 (96 h) (growth)	33.6 µg L^{-1}				33.6	
	Chlorophyta (Chlorophyceae)	*Pseudokirchneriella subcapitata*	EC50 (96h)	64 mg L^{-1}				64 000	[1]
			NOEC (96 h) (growth)	>100 µg L^{-1}				>2 000	[23]
			EC50 (96 h) (growth)	>100 µg L^{-1}				>100	
			NOEC (96 h) (growth inhibition)	>100 000 µg L^{-1}				>2 000 000	[24]
			LOEC (96 h) (growth inhibition)	>100 000 µg L^{-1}				>2 000 000	
		Desmodesmus subspicatus	EC50 (growth inhibition)	74.0 mg L^{-1}				74 000	[25]
	Heterokontophyta (Bacillariophyceae)	*Cyclotella meneghiniana*	EC50 (96 h) (growth)	31.6 µg L^{-1}				31.6	[23]
			NOEC (96 h) (growth)	10 µg L^{-1}				200	[23]
Invertebrate	Arthropoda, Crustacea (Malacostraca)	*Gammarus pulex*	LOEC (behaviour)	10 ng L^{-1}				0.2	[26]
	Arthropoda, Crustacea (Branchiopoda)	*Thamnocephalus platyurus*	LC50 (24 h) (mortality)	> 100 mg L^{-1}				>100 000	[27]
		Ceriodaphnia dúbia (Water flea)	EC50 (48h) (mortality)	77.7 µg L^{-1}				77.7	[23]
					NOEC (7d) (reproduction)	25 µg L^{-1}		2 500	

Therapeutic group/ Pharmaceutical	Phylum(Class)	Specie (common name)	Acute toxicological endpoint	Acute ecotoxicity data	Chronic toxicological endpoint	Chronic ecotoxicity data	Other remarks	PNEC (ng L^{-1})	Reference
			EC50 (48 h) (immobilization)	77 700 µg L^{-1}				77 700	[24]
					NOEC (7d) (reproduction)	25 µg L^{-1}		2 500	
		Daphnia magna (Water flea)	EC50 (48h) (mortality)	>13.8 µg L^{-1}				>13.8	[23]
			EC50 (48 h) (immobilization)	>100 mg L^{-1}				>100 000	[28]
			EC50 (96 h) (immobilization)	76.3 mg L^{-1}				76 300	
			EC50 (48 h) (immobilization)	> 13 800 µg L^{-1}				>13 800	[24]
			EC50 (48 h) (immobilization)	>100 mg L^{-1}				>100 000	[25]
			EC50 (48h) (immobility)	97.8 mg L^{-1}				97 800	[1]
			EC50 (48h) (immobility)	55 mg L^{-1}				55 000	
Fish	Chordata (Actinopterygii)	*Oryzias latipes* (Japanese rice fish)	LC50 (96 h) (mortality)	45.87 mg L^{-1}				45 870	[27]
			LC50 (48 h) (mortality)	35.4 mg L^{-1}				35 400	[28]
			LC50 (96 h) (mortality)	35.4 mg L^{-1}				35 400	
			LC50 (96h) (mortality)	20 mg L^{-1}				20 000	[1]
		Danio rerio (zebrafish)-embryo			NOEC (10 d) (mortality)	25 000 µg L^{-1}		2 500 000	[24]
**SSRIs**									
**Citalopram (CIT)**								**PNEC (ng L^{-1})** **Algae –** 1 600 **Invertebrate –** 3 900 **Fish –** 4 470	

**Therapeutic group/ Pharmaceutical**	**Phylum(Class)**	**Specie (common name)**	**Acute toxicological endpoint**	**Acute ecotoxicity data**	**Chronic toxicological endpoint**	**Chronic ecotoxicity data**	**Other remarks**	**PNEC (ng L^{-1})**	**Reference**
Algae	Chlorophyta (Chlorophyceae)	*Pseudokirchneriella subcapitata* (green microalgae)	EC50 (48 h) (biomass)	1.6 mg L^{-1}				1 600	[29]
Invertebrate	Arthropoda, Crustacea (Branchiopoda)	*Ceriodaphnia dubia* (Water flea)	LC50 (48 h) (survival)	3.90 mg L^{-1}	NOEC (8 d) (neonates produced)<br>LOEC (8 d) (neonates produced)	0.80 mg L^{-1}<br>4.00 mg L^{-1}		3 900<br>80 000<br>400 000	[30]
		Daphnia magna (Water flea)	EC50 (48 h) (biomass)	20 mg L^{-1}				20 000	[29]
	Mollusca (Gastropoda)	*Chlorostoma funebrali*	LOEC (4 h) (adhesion to substract)	405 µg L^{-1}				8 100	[31]
		Lithopoma americanum	LOEC (4 h) (adhesion to substract)	405 µg L^{-1}				8 100	[31]
		Tegula fasciatus	LOEC (4 h) (adhesion to substract)	405 µg L^{-1}				8 100	[31]
		Nucella ostrina	LOEC (4 h) (adhesion to substract)	4.05 mg L^{-1}				81 000	[31]
Fish			LC50 (96 h)	4.47 mg L^{-1}			ECOSAR 2.0	4 470	
**Fluoxetine (FLU)**								**PNEC (ng L^{-1})**<br>**Algae –** 44.99 **Invertebrate –** 2 **Fish –** 2.8	
Algae	Chlorophyta (Chlorophyceae)	*Pseudokirchneriella subcapitata*			EC50 (120 h)	24 µg L^{-1}<br>39 µg L^{-1}		240<br>390	[32,33]

Therapeutic group/ Pharmaceutical	Phylum(Class)	Specie (common name)	Acute toxicological endpoint	Acute ecotoxicity data	Chronic toxicological endpoint	Chronic ecotoxicity data	Other remarks	PNEC (ng L^{-1})	Reference
		(green microalgae)			(growth - turbidity) (growth – cell density)				
			IC50 (96 h) (growth inhibition)	44.99 µg L^{-1}				44.99	[34]
			EC50 (24 h) (growth rate)	90 µg L^{-1}				90	[35]
		Scendesmus acutus (freshwater green microalgae)	IC50 (96 h) (growth inhibition)	91.23 µg L^{-1}				91.23	[34]
		Scendesmus quadricauda (freshwater green microalgae)	IC50 (96 h) (growth inhibition)	212.98 µg L^{-1}				212.98	[34]
		Scendesmus vacuolatus (freshwater green microalgae)	EC50 (24 h) (cell volume growth)	93 µg L^{-1}				93	[35]
		Chlorella vulgaris (single-cell green algae)	IC50 (96 h) (growth inhibition)	4339.25 µg L^{-1}				4339.25	[34]
		Dunaliella tertiolecta	EC50 (96 h) (population cell density)	169.81 µg L^{-1}				169.81	[20]
Invertebrate	Arthropoda, Crustacea (Branchiopoda)	*Ceriodaphnia dubia* – arthropod, crustacean (Water flea)	LC50 (48 h) (survival)	234 µg L^{-1}	NOEC (7 d) (reproduction)<br>LOEC (7 d) (reproduction)	56 µg L^{-1}<br>112 µg L^{-1}		234<br>5 600<br>11 200	[32,33]
			LC50 (48 h) (survival)	0.51 mg L^{-1}		0.089 mg L^{-1}		510<br>8 900	[30]

Therapeutic group/ Pharmaceutical	Phylum(Class)	Specie (common name)	Acute toxicological endpoint	Acute ecotoxicity data	Chronic toxicological endpoint	Chronic ecotoxicity data	Other remarks	PNEC (ng L^{-1})	Reference
					NOEC (8 d) (neonates produced)<br>LOEC (8 d) (neonates produced)	0.447 mg L^{-1}		44 700	
		Daphnia magna (Water flea)	LC50 (48 h) (survival)	820 µg L^{-1}				820	[32,33]
					LOEC (21 d) (reproduction)<br>NOEC (21 d) (reproduction)	429 µg L^{-1}(*R*-Fluoxetine)<br>430µg L^{-1} (Racemic)<br>444 µg L^{-1} (*S*-Fluoxetine)<br>170 µg L^{-1} (*R*-Fluoxetine)<br>174 µg L^{-1} (Racemic)<br>195 µg L^{-1} (*S*-Fluoxetine)		42 900<br>43 000<br>44 400<br>17 000<br>17 400<br>19 500	[36]
					LOEC (21 d) (new bornes lenght)<br>NOEC (21 d) (new bornes lenght)	31 µg L^{-1}<br>8.9 µg L^{-1}		3 100<br>890	[37]
		Thamnocephalus platyurus	LC50 (24 h) (lethality test)	0.76 mg L^{-1}				760	[38]
	Arthropoda, Crustacea (Malacostraca)	*Hyalella azteca*			EC50 (42 d) (survival)	>43 mg kg^{-1}		>430 000 ng kg^{-1}	[32,33]

Therapeutic group/ Pharmaceutical	Phylum(Class)	Specie (common name)	Acute toxicological endpoint	Acute ecotoxicity data	Chronic toxicological endpoint	Chronic ecotoxicity data	Other remarks	PNEC (ng L^{-1})	Reference
					LOEC (42 d) (growth)	5.4 mg kg^{-1}		540 000 ng kg^{-1}	
					LOEC (28 d) (growth)<br>NOEC (28 d) (growth)	100 µg L^{-1}<br>33 µg L^{-1}		10 000<br>3 300	[37]
		Gammarus pulex	LOEC (1.5 h) (activity)	100 ng L^{-1}				2	[26]
	Arthropoda (Insecta)	*Chironomus tentans* (midge)			LC50 (10 d) (survival)<br>LOEC (10 d) (growth)	15.2 mg kg^{-1}<br>1.3 mg kg^{-1}		152 000 ng kg^{-1}<br>130 000 ng kg^{-1}	[32,33]
	Ciliophora (Heterotrichea)	*Spirostomum ambiguum*	EC50 (24 h) (deformity)<br>LC50 (24 h) (deformity)	0.41 mg L^{-1}<br>0.55 mg L^{-1}				410<br>550	[38]
	Mollusca (Gastropoda)	*Potamopyrgus antipodarum* (New Zealand mudsnail)			EC10 (56 d) (embryos without shell)<br>NOEC (56 d) (embryos without shell)	0.81 µg L^{-1}<br>0.47 µg L^{-1}		8.1<br>47	[39]
					LOEC (28 d) (reproduction)<br>NOEC (28 d) (reproduction)	69 µg L^{-1}<br>13 µg L^{-1}		6 900<br>1 300	[37]
					LOEC (42 d) (number of neonates)	100 µg L^{-1}		10 000	[40]

Therapeutic group/ Pharmaceutical	Phylum(Class)	Specie (common name)	Acute toxicological endpoint	Acute ecotoxicity data	Chronic toxicological endpoint	Chronic ecotoxicity data	Other remarks	PNEC (ng L^{-1})	Reference
					NOEC (42 d) (number of neonates)	33.3 µg L^{-1}		3 330	
		Valvata piscinalis (European valve snail)			NOEC (42 d) (cumulate number of eggs)	100 µg L^{-1}		10 000	[40]
		Chlorostoma funebralis – mollusk, gastropod	LOEC (4 h) (adhesion to substract)	345 µg L^{-1}				6 900	[31]
		Lithopoma americanum	LOEC (4 h) (adhesion to substract)	3.45 mg L^{-1}				69 000	[31]
		Tegula fasciatus	LOEC (4 h) (adhesion to substract)	34.5 µg L^{-1}				690	[31]
		Urosalpinx cinérea	LOEC (4 h) (adhesion to substract)	3.45 mg L^{-1}				69 000	[31]
		Nucella ostrina	LOEC (4 h) (adhesion to substract)	3.45 mg L^{-1}				69 000	[31]
	Mollusca (Bivalvia)	*Lampsilis siliquoidea and Ligumia recta* – mollusk, freshwater mussels glochidia juvenile adult female	EC50 (24 h) (valve closure)<br>EC50 (48 h) (foot movement or heartbeat)	239 – 624.8 µg L^{-1}<br>179 – 265.7 µg L^{-1}	LOEC (28 d) (foot protrusion, mantle lure display and	29.3 – 300 µg L^{-1}		239 – 624.8<br>179 – 265.7<br>2 930 - 30 000	[41]

Therapeutic group/ Pharmaceutical	Phylum(Class)	Specie (common name)	Acute toxicological endpoint	Acute ecotoxicity data	Chronic toxicological endpoint	Chronic ecotoxicity data	Other remarks	PNEC (ng L^{-1})	Reference
			EC50 (96 h) (foot movement or heartbeat)	62.0 – 96.9 µg L^{-1}	glochidia parturition)			62.0 – 96.9	
Fish	Chordata (Actinopterygii)	*Pimephales promelas* – fish (fathead minnow)	LC50 (48 h) (toxicity test)	705 µg L^{-1}				705	[32,33]
			LC50 (48 h) (R-,rac-,and S-fluoxetine)	212 µg L^{-1} (*R*-Fluoxetine)				212	[36]
				198 µg L^{-1} (Racemic)				198	
				216 µg L^{-1} (*S*-Fluoxetine)				216	
					LOEC (7 d) (growth)	170 µg L^{-1} (*R*-Fluoxetine)		17 000	
						53 µg L^{-1} (Racemic)		5 300	
						51 µg L^{-1} (*S*-Fluoxetine)		5 100	
					NOEC (7 d) (growth)	118 µg L^{-1} (*R*-Fluoxetine)		11 800	
						9 µg L^{-1} (Racemic)		900	
						9 µg L^{-1} (*S*-Fluoxetine)		900	
					LOEC (21d) (induced vitellogenin in male fish)	28 ng L^{-1}		2.8	[39]
		PLHC-1 *(Poeciliopsis*	EC50 (24 h) (MTT assay - The MTT assay is based on the	6.34 mg L^{-1}<br>3.31 mg L^{-1}				6 340<br>3 310	[42]

Therapeutic group/ Pharmaceutical	Phylum(Class)	Specie (common name)	Acute toxicological endpoint	Acute ecotoxicity data	Chronic toxicological endpoint	Chronic ecotoxicity data	Other remarks	PNEC (ng L^{-1})	Reference
		lucida hepatoma cell)<br>RTG-2 (rainbow trout gonadal cell line)	uptake of thiazolyl blue tetra-zolium bromide (MTT) and its following reduction in the mitochondria of living cells to MTT formazan)						
		Gambusia affinis (mosquitofish)			LC50 (7 d) (Mortality)	546 µg L^{-1}		5 460	[43]
		Oryzias latipes (Japanese medaka)	LC50 (96 h) (bioaccumulation test)<br><br>NOEC (96 h) (bioaccumulation test)	5.5, 1.3, and 0.20 mg L^{-1} at pH 7, 8, and 9, respectively<br><br>3.8 mg L^{-1} at pH 7.1				5 500, 1 300, 200<br><br>3 800	[44]
Other vertebrates	Chordata (Amphibia)	*Cophixalus riparius*-amphibian			LOEC (28 d) (emergence)	1.12 mg kg^{-1}		112 000 ng kg^{-1}	[45]
		Xenopus laevis	EC10 (96 h) (deformity)<br>EC50 (96 h) (deformity)<br>LC10 (96 h) (deformity)<br>LC50 (96 h) (deformity)<br>NOEC (deformity)	3.0 mg L^{-1}<br>4.9 mg L^{-1}<br>7.1 mg L^{-1}<br>7.5 mg L^{-1}<br>2.0 mg L^{-1}				3 000<br>4 900<br>7 100<br>7 500<br>40 000	[46]

Therapeutic group/ Pharmaceutical	Phylum(Class)	Specie (common name)	Acute toxicological endpoint	Acute ecotoxicity data	Chronic toxicological endpoint	Chronic ecotoxicity data	Other remarks	PNEC (ng L^{-1})	Reference
**Norfluoxetine (Nor-FLU)**								**PNEC (ng L^{-1})** **Algae –** 189 **Invertebrate –** 300 **Fish –** n.a	
Algae	(n.a)	(n.a)	EC50 (24 h) (growth rate)	242 µg L^{-1}				242	[35]
	(n.a)	(n.a)	EC50 (24 h) (cell volume growth)	189 µg L^{-1}				189	[35]
Invertebrate	Arthropoda, Crustacea (Branchiopoda)	*Thamnocephalus platyurus*	LC50 (24 h) (deformity)	0.47 mg L^{-1}				470	[38]
	Ciliophora (Heterotrichea)	*Spirostomum ambiguum*	EC50 (24 h) (deformity)	0.30 mg L^{-1}				300	[38]
			LC50 (24 h) (deformity)	0.39 mg L^{-1}				390	
**Paroxetine (PAR)**								**PNEC (ng L^{-1})** **Algae –** 140 **Invertebrate –** 580 **Fish –** 3 290	
Algae	Chlorophyta (Chlorophyceae)	*Pseudokirchneriella subcapitata* (green microalgae)	EC50 (48 h) (biomass)	0.14 mg L^{-1}				140	[29]
Invertebrate	Arthropoda, Crustacea (Branchiopoda)	*Ceriodaphnia dubia* (Water flea)	LC50 (48 h) (survival)	0.58 mg L^{-1}				580	[30]
					NOEC (8 d) (neonates produced)	0.22 mg L^{-1}		22 000	
					LOEC (8 d) (neonates produced)	0.44 mg L^{-1}		44 000	
		Daphnia magna (Water flea)	EC50 (48 h) (biomass)	6.3 mg L^{-1}				6 300	[29]
			EC 50 (48 h) (immobilization)	35.0 mg L^{-1}				35 000	[47]

Therapeutic group/ Pharmaceutical	Phylum(Class)	Specie (common name)	Acute toxicological endpoint	Acute ecotoxicity data	Chronic toxicological endpoint	Chronic ecotoxicity data	Other remarks	PNEC (ng L^{-1})	Reference
Fish			LC50 (96h)	3.29 mg L^{-1}			ECOSAR 2.0	3 290	
Other vertebrates	Chordata (Amphibia)	*Xenopus laevis*- amphibian	EC10 (96 h) (deformity)	3.6 mg L^{-1}				3 600	[46]
			EC50 (96 h) (deformity)	4.1 mg L^{-1}				4 100	
			LC10 (96 h) (deformity)	4.4 mg L^{-1}				4 400	
			LC50 (96 h) (deformity)	5.12 mg L^{-1}				5 120	
			NOEC (deformity)	2.0 mg L^{-1}				40 000	
**Sertraline (SER)**								**PNEC (ng L^{-1})** **Algae –** 12.10 **Invertebrate –** 120 **Fish –** 72	
Bacteria	Proteobacteria (Gammaproteobacteria)	*Vibrio fischeri*	EC50 (30 min) (inhibition)	10.72 mg L^{-1}				10 720	[48]
			NOEC (30 min) (inhibition)	2.25 mg L^{-1}				45 000	
			LOEC (30 min) (inhibition)	4.5 mg L^{-1}				90 000	
Algae	Chlorophyta (Chlorophyceae)	*Scendesmus acutus* (freshwater green microalgae)	IC50 (96 h) (growth inhibition)	98.92 µg L^{-1}				98.92	[34]
		Scendesmus quadricauda (freshwater green microalgae)	IC50 (96 h) (growth inhibition)	317.02 µg L^{-1}				317.02	[34]
		Chlorella vulgaris (single-cell green algae)	IC50 (96 h) (growth inhibition)	763.66 µg L^{-1}				763.66	[34]
		Pseudokirchneriella subcapitata	EC50 (48 h) (biomass)	0.043 mg L^{-1}				43	[29]

Therapeutic group/ Pharmaceutical	Phylum(Class)	Specie (common name)	Acute toxicological endpoint	Acute ecotoxicity data	Chronic toxicological endpoint	Chronic ecotoxicity data	Other remarks	PNEC (ng L^{-1})	Reference
		(green microalgae)	IC50 (96 h) (growth inhibition)	12.10 µg L^{-1}				12.10	[34]
			EC50 (72 h) (inhibition)	0.14 mg L^{-1}				140	[48]
			NOEC (72 h) (inhibition)	0.05 mg L^{-1}				1000	
			LOEC (72 h) (inhibition)	0.075 mg L^{-1}				1 500	
Invertebrate	Arthropoda, Crustacea (Branchiopoda)		LC50 (48 h) (survival)	0.12 mg L^{-1}				120	[30]
					NOEC (8 d) (neonates produced)	0.009 mg L^{-1}		900	
					LOEC (8 d) (neonates produced)	0.045 mg L^{-1}		4 500	
		Ceriodaphnia dubia (Water flea)	EC50 (48 h) (offspring)	126 µg L^{-1}				126	[49]
					LOEC (1st and 2nd generations) (fecundity and growth)	53.4 µg L^{-1}		5 340	
					LOEC (3rd generation) (fecundity and growth)	4.8 µg L^{-1}		480	
					EC50 (mean) (offspring)	17.2 µg L^{-1}		172	
		Daphnia magna (Water flea)	EC50 (48 h) (biomass)	0.92 mg L^{-1}				920	[29]
			EC50 (48 h) (immobilization)	1.3 mg L^{-1}				1 300	[48]

Therapeutic group/ Pharmaceutical	Phylum(Class)	Specie (common name)	Acute toxicological endpoint	Acute ecotoxicity data	Chronic toxicological endpoint	Chronic ecotoxicity data	Other remarks	PNEC (ng L^{-1})	Reference
			NOEC (24 h) (immobilization)	0.10 mg L^{-1}				2 000	
			LOEC (24 h) (immobilization)	0.18 mg L^{-1}				3 600	
					EC50 (21 d) (reproduction)	0.066 mg L^{-1}		660	
					NOEC (21 d) (reproduction)	0.032 mg L^{-1}		3 200	
					LOEC (21 d) (reproduction)	0.1 mg L^{-1}		10 000	
					EC50 (21 d) (lethality)	0.12 mg L^{-1}		1 200	
					NOEC (21 d) (lethality)	0.032 mg L^{-1}		3 200	
					LOEC (21 d) (lethality)	0.1 mg L^{-1}		10 000	
		Thamnocephalus platyurus	LC50 (24 h) (lethality)	0.6 mg L^{-1}				600	[48]
			NOEC (24 h) (lethality)	0.4 mg L^{-1}				8 000	
			LOEC (24 h) (lethality)	0.6 mg L^{-1}				12 000	
Fish	Chordata (Actinopterygii)	*Oncorhynchus mykis*	LC50 (96 h) (lethality)	0.38 mg L^{-1}				380	[48]
			NOEC (96 h) (lethality)	0.1 mg L^{-1}				2 000	
			LOEC (96 h) (lethality)	0.32 mg L^{-1}				6 400	
		Pimephales promelas- fish (fathead minnow)	LC50 (48 h) pH 6.5 (lethality)	647 µg L^{-1}				647	[50]
			LC50 (48 h) pH 7.5 (lethality)	205 µg L^{-1}				205	

Therapeutic group/ Pharmaceutical	Phylum(Class)	Specie (common name)	Acute toxicological endpoint	Acute ecotoxicity data	Chronic toxicological endpoint	Chronic ecotoxicity data	Other remarks	PNEC (ng L^{-1})	Reference
			LC50 (48 h) pH 8.5 (lethality)	72 µg L^{-1}				72	
					EC50 (7 d) (growth, survival) pH 6.5	544.4 µg L^{-1}		5 444	
					EC50 (7 d) (feeding rate) pH 6.5	199.7 µg L^{-1}		1 997	
					EC50 (7 d) (growth, survival) pH 7.5	131.4 µg L^{-1}		1 314	
					EC50 (7 d) (feeding rate) pH 7.5	149.5 µg L^{-1}		1 495	
					EC50 (7 d) (growth, survival) pH 8.5	50 µg L^{-1}		500	
					EC50 (7 d) (feeding rate) pH 8.5	80.3 µg L^{-1}		803	
	Chordata (Amphibia)	*Xenopus laevis* - amphibian	EC10 (96 h) (deformity)	3.0 mg L^{-1}				3 000	[46]
			EC50 (96 h) (deformity)	3.3 mg L^{-1}				3 300	
			LC10 (96 h) (deformity)	3.6 mg L^{-1}				3 600	
			LC50 (96 h) (deformity)	3.9 mg L^{-1}				3 900	
			NOEC (deformity)	1.0 mg L^{-1}				20 000	
**Anti-inflammatories**									
**Diclofenac (DIC)**								**PNEC (ng L^{-1})** **Algae –** 200 **Invertebrate –** 20 000 **Fish –** 50	

Therapeutic group/ Pharmaceutical	Phylum(Class)	Specie (common name)	Acute toxicological endpoint	Acute ecotoxicity data	Chronic toxicological endpoint	Chronic ecotoxicity data	Other remarks	PNEC (ng L^{-1})	Reference
Bacteria	Proteobacteria (Gammaproteobacteria)	*Vibrio fischeri*	EC50 (30 min) (luminescence)	11 454 µg L^{-1}				11 454	[24]
			EC50 (inhibition of luminescence)	13 500 µg L^{-1}<br>13 700 µg L^{-1}			Two test were assayed ToxAlert 100 Microtox	13 500<br>13 700	[17]
			EC50 (30 min) Luminescence	11 454 µg L^{-1}				11 454	[23]
Algae	Cyanobacteria, Synechococcales	*Synechococcus leopolensis*	EC50 (96 h) (growth)<br>NOEC (96 h) (growth)	14 500 µg L^{-1}<br>10 000 µg L^{-1}				14 500<br>200 000	[23]
			LOEC (growth)	10 µg L^{-1}			Test performed in river biofilm community	200	[51]
	Chlorophyta (Chlorophyceae)	*Desmodesmus subspicatus*	EC50 (algal growth inhibition test)	72 mg L^{-1}				72 000	[25]
		Dunaliella tertiolecta	EC50 (96 h) (growth inhibition)	185 690 µg L^{-1}				185 690	[20]
		Raphidocelis subcapitata (Selenastrum capricornutum, Pseudokirchneriella subcapitata)	NOEC (96 h) (growth inhibition)<br>LOEC (96 h) (growth inhibition)	10 000 µg L^{-1}<br>20 000 µg L^{-1}				200 000<br>400 000	[24]
			EC50 (96 h) (growth)	16 300 µg L^{-1}				16 300	[23]

Therapeutic group/ Pharmaceutical	Phylum(Class)	Specie (common name)	Acute toxicological endpoint	Acute ecotoxicity data	Chronic toxicological endpoint	Chronic ecotoxicity data	Other remarks	PNEC (ng L^{-1})	Reference
			NOEC (96 h) (growth)	10 000 µg L^{-1}				200 000	[23]
	Bacillariophyta (Coscinodiscophyceae)	*Cyclotella meneghiniana*	EC50 (96 h) (growth)	19 240 µg L^{-1}				19 240	[23]
			NOEC (96 h) (growth)	10 000 µg L^{-1}				200 000	[23]
	Tracheophyta (Liliopsida)	*Lemna minor* (duckweed)	EC50 (7 d) (growth inhibition)	7.5 mg L^{-1}				7 500	[25]
Invertebrate	Arthropoda, Crustacea (Branchiopoda)	*Ceriodaphnia dubia*	EC50 (48 h) (acute immobilisation test)	22 704 µg L^{-1}				22 704	[24]
					NOEC (7 d) (reproduction test)	1 000 µg L^{-1}		100 000	[24]
					LOEC (7 d) (reproduction test)	2 000 µg L^{-1}		200 000	
		Daphnia magna	EC50 (48 h) (acute immobilisation test)	68 mg L^{-1}				68 000	[25]
			EC50 (48 h) (acute immobilisation test)	22 430 µg L^{-1}				22 430	[24]
					EC50 (21 d) (reproduction test)	11.04 mg L^{-1}		110 400	[52]
			LOEC (48 h) (immobilisation)	40 mg L^{-1}				800 000	[53]
			NOEC (48 h) (immobilisation)	30 mg L^{-1}				600 000	

Therapeutic group/ Pharmaceutical	Phylum(Class)	Specie (common name)	Acute toxicological endpoint	Acute ecotoxicity data	Chronic toxicological endpoint	Chronic ecotoxicity data	Other remarks	PNEC (ng L^{-1})	Reference
					NOEC (21 d) (reproduction)<br>LOEC (21 d) (reproduction)	200 µg L^{-1}<br>1 mg L^{-1}		20 000<br>100 000	[53]
			EC50 (24 h) (mortality)	74.27 mg L^{-1}				74 270	[53]
			EC50 (24 h) (mortality)	56 mg L^{-1}				56 000	[53]
			EC50 (48 h) (mortality)	80.1 mg L^{-1}				80 100	[15]
					LOEC (21 d) (reproduction)	10 mg L^{-1}		1 000 000	[15]
			EC50 (48h) (mortality)	22.4 mg L^{-1}				22 400	[1]
		Thamnocephalus platyurus	LC50 (24 h) (toxicity test with larvae)<br>EC50 (1 h) (toxicity test with larvae)	41.0 mg L^{-1}<br>46.0 mg L^{-1}			Thamnotoxkit<br>Rapidtoxkit	41 000<br>46 000	[54]
	Rotifera (Monogononta)	*Brachionus calyciflorus*			NOEC (48 h) (reproduction test)<br>LOEC (48 h) (reproduction test)	12 500 µg L^{-1}<br>25 000 µg L^{-1}		1 250 000<br>2 500 000	[24]
Fish	Chordata (Actinopterygii)	*Danio rerio* (zebrafish)	EC50 (96h) (teratogenicity)	0.09 mg L^{-1}				90	[1]
			LC50 (96h) (n.a)	0.48 mg L^{-1}				480	[1]

Therapeutic group/ Pharmaceutical	Phylum(Class)	Specie (common name)	Acute toxicological endpoint	Acute ecotoxicity data	Chronic toxicological endpoint	Chronic ecotoxicity data	Other remarks	PNEC (ng L^{-1})	Reference
					NOEC (10 d) (survival)	4 000 µg L^{-1}		400 000	[24]
					LOEC (10 d) (survival)	8 000 µg L^{-1}		800 000	
			EC50 (96 h) (mortality)	214 mg L^{-1}				214 000	[53]
			NOEC (4 d) (hatching rate)	1 131 µg L^{-1}				22 620	[55]
			NOEC (development rate)	1 131 µg L^{-1}				22 620	[55]
					NOEC (34 d) (survival)	320 µg L^{-1}		32 000	[55]
		Oncorhynchus Mykiss (rainbow trout)			NOEC (28 d) (Hsp70 in liver and kidney)	500 µg L^{-1}		50 000	[53]
					LOEC (28 d) (histopathological alterations)	5 µg L^{-1}		500	[56]
					LOEC (28 d) (cytological alterations)	1 µg L^{-1}		100	[57]
					NOEC (95 d) (histopathological effects on gills)	320 µg L^{-1}		32 000	[55]
					LOEC (21 d) (histopathological effects in kidney)	1 µg L^{-1}	(LOEC was not calculated in the paper)	100	[58]

Therapeutic group/ Pharmaceutical	Phylum(Class)	Specie (common name)	Acute toxicological endpoint	Acute ecotoxicity data	Chronic toxicological endpoint	Chronic ecotoxicity data	Other remarks	PNEC (ng L^{-1})	Reference
					LOEC (21 d) (histopathological effects in intestine)	1 µg L^{-1}	(LOEC was not calculated in the paper)	100	[58]
		Salmo trutta (trout)			NOEC (21 d) (histopathological alterations)	0.5 µg L^{-1}	(subchronic exposure)	50	[59]
					NOEC (90 d) LOEC (90 d) (hatching time)	1 mg L^{-1}<br>2 mg L^{-1}		100 000<br>200 000	[53]
					NOEC (90 d) (Hsp70 level)	2 mg L^{-1}		200 000	[53]
					NOEC (60 d) (mortality, hatching, development, teratogenicity)	500 µg L^{-1}		50 000	[53]
Other vertebrates	Chordata (Aves)	*Gyps bengalensis* (Oriental white-backed vulture)			LOAEL (8 d) (dietary intake, renal failure)	0.007 mg kg^{-1}		700 ng kg^{-1}	[60]
**4-hydroxydiclofenac (4-OH-DIC)**									**PNEC (ng L^{-1})** **Algae –** 660 300 **Invertebrate –** 48 200 **Fish –** 65 200
Algae	(n.a)	Algae (n.a)			EC50 (toxicity)	66.03 mg L^{-1}		660 300	[61]
Invertebrates		Daphnid	LC50 (48 h)	48.2 mg L^{-1}			ECOSAR 2.0	48 200	
Fish			LC50 (96 h)	65.2 mg L^{-1}			ECOSAR 2.0	65 200	
**Ibuprofen (IBU)**									**PNEC (ng L^{-1})** **Algae –** 40 100 **Invertebrate –** 0.2 **Fish –** 180

**Therapeutic group/ Pharmaceutical**	**Phylum(Class)**	**Specie (common name)**	**Acute toxicological endpoint**	**Acute ecotoxicity data**	**Chronic toxicological endpoint**	**Chronic ecotoxicity data**	**Other remarks**	**PNEC (ng L^{-1})**	**Reference**
Bacteria	Proteobacteria (Gammaproteobacteria)	*Vibrio fischeri*	EC50 (inhibition of luminescence)	12 100 ng L^{-1}<br>19 100 ng L^{-1}			Two test were assayed ToxAlert 100 Microtox	12 100<br>19 100	[17]
Algae	Chlorophyta (Chlorophyceae)	*Desmodesmus subspicatus*	EC50 (algal growth inhibition test)	315 mg L^{-1}				315 000	[25]
	Tracheophyta (Liliopsida)	*Lemna minor* (duckweed)			EC50 (7 d) (growth inhibition)	22 mg L^{-1}		220 000	[25]
					EC50 (7 d) (growth inhibition)	4.01 mg L^{-1}		40 100	[62]
Invertebrate	Arthropoda, Crustacea (Branchiopoda)	*Daphnia magna*	EC50 (48 h) (acute immobilisation test)	108 mg L^{-1}				108 000	[25]
					EC50 (14 d) (reproduction test)	13.4 mg L^{-1}		134 000	[63]
					NOEC (14 d) LOEC (14 d) (survival)	20 mg L^{-1}<br>80 mg L^{-1}		2 000 000<br>8 000 000	[63]
					LOEC (14 d) (population growth rate)	20 mg L^{-1}		2 000 000	[63]
					EC50 (21 d) (reproduction test)	7.87 mg L^{-1}		78 700	[52]
			EC50 (48 h)	132.6 mg L^{-1}				132 600	[15]

Therapeutic group/ Pharmaceutical	Phylum(Class)	Specie (common name)	Acute toxicological endpoint	Acute ecotoxicity data	Chronic toxicological endpoint	Chronic ecotoxicity data	Other remarks	PNEC (ng L^{-1})	Reference
			(mortality)						
					EC50 (21 d) (reproduction test)	20 mg L^{-1}		200 000	[15]
			EC50 (24 h) (toxicity)	5.7 mg L^{-1}				5 700	[64]
		Thamnocephalus platyurus	LC50 (24 h) (mortality)	19.59 mg L^{-1}				19 590	[27]
	Arthropoda, Crustacea (Malacostraca)	*Gammarus pulex*	LOEC (2 h) (behavioural test)	10 ng L^{-1}				0.2	[26]
	Mollusca (Gastropoda)	*Planorbis carinatus* (freshwater snail)	LC50 (72 h) (survival)	17.1 mg L^{-1}				17 100	[65]
					NOEC (21 d) (survival)	5.36 mg L^{-1}		536 000	
					NOEC (21 d) (growth test)	1.02 mg L^{-1}		102 000	
					LOEC (21 d) (growth test)	2.43 mg L^{-1}		243 000	
					NOEC (21 d) (reproduction test)	2.43 mg L^{-1}		243 000	
	Cnidaria (Hydrozoa)	*Hydra attenuata*	LC50 (96 h) (morphology)	22.36 mg L^{-1}				22 360	[13]
			EC50 (96 h) (morphology)	1.65 mg L^{-1}				1 650	
			LOEC (96 h) (morphology)	1 mg L^{-1}				20 000	
			NOEC (96 h) (morphology)	0.1 mg L^{-1}				2 000	
			EC50 (96 h) (feeding)	3.85 mg L^{-1}				3 850	[13]

Therapeutic group/ Pharmaceutical	Phylum(Class)	Specie (common name)	Acute toxicological endpoint	Acute ecotoxicity data	Chronic toxicological endpoint	Chronic ecotoxicity data	Other remarks	PNEC (ng L^{-1})	Reference
Fish	Chordata (Actinopterygii)	*Oryzias latipes* (Japanese rice fish)	LC50 (96 h) (mortality)	>100 mg L^{-1}				>100 000	[27]
		Carassius auratus			NOEC (7 d) (toxicity)	1.80 µg L^{-1}		180	[66]
**Naproxen (NAP)**								**PNEC (ng L^{-1})** **Algae –** 31 820 **Invertebrate –** 2 620 **Fish –** 115 200	
Algae	Chlorophyta (Chlorophyceae)	*Desmodesmus subspicatus*	EC50 (3 d) (growth inhibition)	320.0 mg L^{-1}				320 000	[25]
			EC50 (growth inhibition)	625.5 mg L^{-1}				625 500	[67]
		Pseudokirchneriella subcapitata	EC50 (72 h) (growth inhibition)	31.82 mg L^{-1}				31 820	[68]
Invertebrate	Arthropoda, Crustacea (Branchiopoda)	*Ceriodaphnia dubia*	EC50 (24h) (immobilization)	66.37 mg L^{-1}				66 370	[68]
					EC50 (7 d) (population growth inhibition)	0.33 mg L^{-1}		3 300	[68]
		Daphnia magna	EC50 (24h) (n.a)	140 mg L^{-1}				140 000	[69]
			EC50 (48h) (immobilization)	174 mg L^{-1}				174 000	[25]
			EC50 (48 h) (immobilization)	166.3 mg L^{-1}				166 300	[67]
		*Daphnia longispina (*cladocera)	LC50 (48 h) (inhibited growth)	82 mg L^{-1}				82 000	[70]
		Thamnocephalus platyurus	LC50 (24h) (Mortality)	84.09 mg L^{-1}				84 090	[68]

Therapeutic group/ Pharmaceutical	Phylum(Class)	Specie (common name)	Acute toxicological endpoint	Acute ecotoxicity data	Chronic toxicological endpoint	Chronic ecotoxicity data	Other remarks	PNEC (ng L^{-1})	Reference
	Ciliophora (Ciliatela)	*Paramecium caudatum* (protozoa)	LC50 (24 h) (inhibited growth)	36 mg L^{-1}				36 000	[70]
	Cnidaria (Hydrozoa)	*Hydra attenuata*	LC50 (96 h) (morphology)	22.36 mg L^{-1}				22 360	[13]
			EC50 (96h) (morphology)	2.62 mg L^{-1}				2 620	
			LOEC (96 h) (morphology)	5 mg L^{-1}				100 000	
			NOEC (96 h) (morphology)	1 mg L^{-1}				20 000	
	Platyhelminthes (Tricladida)	*Dugesia japónica* (planarian)	LC50 (48 h) (n.a)	8.6 mg L^{-1}				8 600	[71]
	Rotifera (Monogononta)	*Brachionu calyciflorus*	LC50 (24h) (Mortality)	62.48 mg L^{-1}				62 480	[68]
					EC50 (48 h) (growth inhibition)	0.56 mg L^{-1}		5 600	
Fish	Chordata (Actinopterygii)	*Lepomis macrochirus*	LC50 (96h) (n.a)	560 mg L^{-1}				560 000	[69]
		Oncorhynchus Mykiss (rainbow trout)	LC50 (96h) (n.a)	690 mg L^{-1}				690 000	[69]
		Danio rerio (zebrafish)	LC50 (96h) (embryos)	115.2 mg L^{-1}				115 200	[72]
			LC50 (96h) (larvae)	147.6 mg L^{-1}				147 600	
**Paracetamol (PARA)**								**PNEC (ng L^{-1})** **Algae –** 134 000 **Invertebrate –** 2 040 **Fish –** 378 000	

Therapeutic group/ Pharmaceutical	Phylum(Class)	Specie (common name)	Acute toxicological endpoint	Acute ecotoxicity data	Chronic toxicological endpoint	Chronic ecotoxicity data	Other remarks	PNEC (ng L^{-1})	Reference
Bacteria	Proteobacteria (Gammaproteobacteria)	*Vibrio fischeri*	EC50 (15 min) (toxicity tests)	567.5 mg L^{-1}				567 500	[28]
			EC50 (30 min) (inhibition of luminescence)	650 mg L^{-1}				650 000	[73]
Algae	Chlorophyta (Chlorophyceae)	*Scenedesmus subspicatus*	EC50 (72 h) (growth inhibition)	134 mg L^{-1}				134 000	[73]
Invertebrate	Arthropoda, Crustacea (Branchiopoda)	*Daphnia magna*	EC50 (96 h) (immobility)	26.6 mg L^{-1}				26 600	[28]
			EC50 (48 h) (immobilisation)	50 mg L^{-1}				50 000	[73]
			EC50 (48 h) (mortality)	20.1 mg L^{-1}				20 100	[15]
			EC50 (24 h) (toxicity)	2.04 mg L^{-1}				2 040	[64]
		Thamnocephalus platyurus	LC50 (24 h) (toxicity tests)	63.8 mg L^{-1}			Thamnotoxkit	63 800	[54]
	Ciliophora (Oligohymenophorea)	*Tetrahymena pyriformis*	EC50 (48 h) (growth inhibition)	112 mg L^{-1}				112 000	[73]
Fish	Chordata (Actinopterygii)	*Oryzias latipes* (Japanese rice fish)	EC50 (96 h) (mortality)	>160 mg L^{-1}				>160 000	[28]
		Danio rerio (zebrafish)	LC50 (48 h) (fish embryos pulse rate)	378 mg L^{-1}				378 000	[73]
			LC50 (48 h) (fish embryos pulse rate)	920 mg L^{-1}				920 000	
**4-aminophenol (4-PARA)**								**PNEC (ng L^{-1})** **Algae –** 11 300 **Invertebrate –** 240 **Fish –** 1 430	
Algae		Green algae	EC50 (96h)	11.3 mg L^{-1}			ECOSAR 2.0	11 300	

Therapeutic group/ Pharmaceutical	Phylum(Class)	Specie (common name)	Acute toxicological endpoint	Acute ecotoxicity data	Chronic toxicological endpoint	Chronic ecotoxicity data	Other remarks	PNEC (ng L^{-1})	Reference
Invertebrate	Arthropoda, Crustacea (Branchiopoda)	*Daphnia magna* (Water flea)	EC50 (24 h)	0.31 mg L^{-1}				310	[74]
			EC50 (48 h)	0.24 mg L^{-1}				240	
Fish	Chordata (Actinopterygii)	*Danio rerio* (zebrafish)	LC50 (72h) embryo mortality	1.43 mg L^{-1}				1 430	[75]
			LC50 (96h) (n.a)	1.44 mg L^{-1}				1 440	[75]
**Hormones**									
**Estrone (E1)**								**PNEC (ng L^{-1})** **Algae –** 355 **Invertebrate –** 3 160 **Fish –** 3.4	
Algae		Green algae	EC50 (96 h)	0.355 mg L^{-1}			ECOSAR 2.0	355	
Invertebrate	Platyhelminthes (Tricladida)	*Dugesia japónica* (planarian)	LC50 (24 h) (toxicity)	>50 mg L^{-1}				>50 000	[76]
			(48 h) (toxicity)	>50 mg L^{-1}				>50 000	
			(72 h) (toxicity)	>50 mg L^{-1}				>50 000	
			(96 h) (toxicity)	>50 mg L^{-1}				>50 000	
		Daphnid	LC50 (48 h)	3.16 mg L^{-1}			ECOSAR 2.0	3 160	
Fish	Chordata (Actinopterygii)	*Oryzias latipes* (Japanese rice fish)			NOEC (100 d) (feminization)	100 ng L^{-1}	NOEC 10 for testis–ova	10	[77]
					NOEC (239 d) (time to hatch egg)	198 ng L^{-1}		19.8	[78]
					LOEC (239 d) (time to hatch egg)	494 ng L^{-1}		49.8	
					NOEC (239 d) (fertility)	484 ng L^{-1}		48.4	

Therapeutic group/ Pharmaceutical	Phylum(Class)	Specie (common name)	Acute toxicological endpoint	Acute ecotoxicity data	Chronic toxicological endpoint	Chronic ecotoxicity data	Other remarks	PNEC (ng L^{-1})	Reference
					LOEC (239 d) (fertility)	1 188 ng L^{-1}		118.8	
		Pimephales promelas (fathead minnow)			LOEC (21 d) (vitellogenin induction)	34 ng L^{-1}		3.4	[79]
**17β-estradiol (E2)**								**PNEC (ng L^{-1})** **Algae –** 162 **Invertebrate –** 1 500 **Fish –** 0.29	
Algae		Green algae	EC50 (96 h)	0.162 mg L^{-1}			ECOSAR 2.0	162	
Invertebrate	Platyhelminthes (Tricladida)	*Dugesia japónica* (planarian)	LC50 (24 h) (toxicity)<br>(48 h) (toxicity)<br>(72 h) (toxicity)<br>(96 h) (toxicity)	> 5 mg L^{-1}<br>2.6 mg L^{-1}<br>2.0 mg L^{-1}<br>1.5 mg L^{-1}				>5 000<br>2 600<br>2 000<br>1 500	[76]
	Arthropoda, Crustacea (Branchiopoda)	*Daphnia magna* (Water flea)	LC50 (48 h) (mobility)	2.87 mg L^{-1}				2 870	[76]
Fish	Chordata (Actinopterygii)	*Danio rerio* (zebrafish)			NOEC (21 d) (Gonadosomatic index (GSI))<br>LOEC (21 d) (Gonadosomatic index (GSI))	25 ng L^{-1}<br>86 ng L^{-1}		2.5<br>8.6	[80]
					NOEC (21 d) (Reproduction)<br>LOEC (21 d) (Reproduction)	5 ng L^{-1}<br>25 ng L^{-1}		0.5<br>2.5	[81]

Therapeutic group/ Pharmaceutical	Phylum(Class)	Specie (common name)	Acute toxicological endpoint	Acute ecotoxicity data	Chronic toxicological endpoint	Chronic ecotoxicity data	Other remarks	PNEC (ng L^{-1})	Reference
					NOEC (21 d) (Egg production and fertility hatching)	27 ng L^{-1}		2.7	[82]
					LOEC (21 d) (Egg production and fertility hatching)	87 ng L^{-1}		8.7	
					NOEC (210 d) (Reproduction)	> 5 ng L^{-1}		>0.5	[83]
		Cyprinodon variegatus (Sheepshead minnow)			NOEC (280 d) (Multi-gen reproduction)	40 ng L^{-1}		4	[84]
					LOEC (280 d) (Multi-gen reproduction)	80 ng L^{-1}		8	
		Gabiocypris rarus (Chinese rare minnow)			NOEC (21 d) (n.a)	5 ng L^{-1}		0.5	[85]
					LOEC (21 d) (n.a)	5 ng L^{-1}		0.5	
		Melanotaenia fluviatilis (Australian rainbowfish)			NOEC (14 d) (n.a)	100 ng L^{-1}		10	[86]
		Oryzias latipes (Japanese rice fish)			NOEC (100 d) (Feminization)	100 ng L^{-1}		10	[77]

Therapeutic group/ Pharmaceutical	Phylum(Class)	Specie (common name)	Acute toxicological endpoint	Acute ecotoxicity data	Chronic toxicological endpoint	Chronic ecotoxicity data	Other remarks	PNEC (ng L^{-1})	Reference
					LOEC (21 d) (testis-ova induction)	< 26.3 ng L^{-1}		<2.63	[87]
					NOEC (21 d) (VTG induction)	29.3 ng L^{-1}		2.93	
					NOEC (21 d) (Papillary processes)	85 ng L^{-1}		8.5	[80]
					NOEC (21 d) (F0 egg production and fertility F1 hatchability)	29.3 ng L^{-1}		2.93	[87]
					LOEC (21 d) (F0 egg production and fertility F1 hatchability)	227 ng L^{-1}		22.7	
					NOEC (20 d) (Reproduction)	34 ng L^{-1}		3.4	[88]
					NOEC (101 d) (Feminization, egg production, fertility, fertility, hatch)	2.9 ng L^{-1}		0.29	[89]
					LOEC (101 d) (Feminization, egg production,	8.7 ng L^{-1}		0.87	

Therapeutic group/ Pharmaceutical	Phylum(Class)	Specie (common name)	Acute toxicological endpoint	Acute ecotoxicity data	Chronic toxicological endpoint	Chronic ecotoxicity data	Other remarks	PNEC (ng L^{-1})	Reference
					fertility, fertility, hatch)				
					NOEC (14 d) (Egg production, fecundity, spawning, fertility hatch)	379 ng L^{-1}		37.9	[90]
		Oryziaz javanicus (Javanese rice fish)			NOEC (187 d) (Fertilization, egg number)	16 ng L^{-1}		1.6	[91]
					LOEC (187 d) (Fertilization, egg number)	16 ng L^{-1}		1.6	
		Pimephales promelas (fathead minnow)			NOEC (29 d) (ELS, growth)	83 ng L^{-1}		8.3	[92]
					NOEC (92 d) (Development)	8 ng L^{-1}		0.8	[92]
					NOEC (46 d) (PLC eggs/female, hatch)	26 ng L^{-1}		2.6	[92]
					NOEC (19 d) (Egg production)	6.6 ng L^{-1}		0.66	[93]
					LOEC (19 d) (Egg production)	30 ng L^{-1}		3	
					NOEC (21 d) (n.a)	9 ng L^{-1}		0.9	[94]

Therapeutic group/ Pharmaceutical	Phylum(Class)	Specie (common name)	Acute toxicological endpoint	Acute ecotoxicity data	Chronic toxicological endpoint	Chronic ecotoxicity data	Other remarks	PNEC (ng L^{-1})	Reference
					LOEC (21 d) (n.a)	44 ng L^{-1}		4.4	
					NOEC (21 d) (Nuptial tubercles)	9 ng L^{-1}		0.9	[80]
					LOEC (21 d) (Nuptial tubercles)	29 ng L^{-1}		2.9	
		Poecilia reticulate (Guppy)			NOEC (90 d) (Feminization)	100 ng L^{-1}		10	[95]
		Pomatoschistus minutus (Sand goby)			NOEC (240 d)	16 ng L^{-1}		1.6	[96]
					LOEC (240 d)	16 ng L^{-1}		1.6	
**17α-ethinylestradiol (EE2)**								**PNEC (ng L^{-1})** **Algae –** 730 **Invertebrate –** 10 **Fish –** 0.01	
Algae	Chlorophyta (Chlorophyceae)	*Pseudokirchneriella subcapitata* (Korshikov)	EC50 (96 h) (growth inhibition)	800 µg L^{-1}				800	[97]
			NOEC (96 h) (growth rate)	< 10 µg L^{-1}				<200	
		Desmodesmus subspicatus	EC50 (96 h) (growth inhibition)	730 µg L^{-1}				730	[97]
			NOEC (96 h) (growth rate)	< 10 µg L^{-1}				<200	
Invertebrate	Arhtropoda, Crustacea (Branchiopoda)	*Ceriodaphnia reticulata*	EC50 (24 h) (swim inhibition)	1 814 µg L^{-1}				1 814	[98]
		Sida crystallina	EC50 (24 h) (swim inhibition)	>4 100 µg L^{-1}				>4 100	[98]
			EC50 (24 h) (n.a)	5.7 mg L^{-1}				5 700	[98]

Therapeutic group/ Pharmaceutical	Phylum(Class)	Specie (common name)	Acute toxicological endpoint	Acute ecotoxicity data	Chronic toxicological endpoint	Chronic ecotoxicity data	Other remarks	PNEC (ng L^{-1})	Reference
		Daphnia magna (Water flea)			NOEC (21 d) (reproduction)	≥100 000 ng L^{-1}		≥10 000	[99]
	Arthropoda, Crustacea (Malacostraca)	*Hyalella azteca*			NOEC (273 d) (reproduction)	100 ng L^{-1}		10	[100]
	Mollusca (Gastropoda)	*Potamopyrgus antipodarum*			NOEC (63 d) (Embryo production)	100 ng L^{-1}		10	[101]
Fish	Chordata (Actinopterygii)	*Cyprinodon variegatus* (Sheepshead minnow)			NOEC (59 d) (Reproduction)	18 ng L^{-1}		1.8	[102]
		Danio rerio (zebrafish)			NOEC (210 d) (Reproduction)<br>LOEC (210 d) (Reproduction)	0.5 ng L^{-1}<br>5 ng L^{-1}		0.05<br>0.5	[83]
					LOEC (38 dph) (plasma VTG induction)	2 ng L^{-1}		0.2	[103]
					NOEC (177 d) (F0 reproduction)<br>NOEC (162 d) (F1 reproduction)	0.31 ng L^{-1}<br>0.36 ng L^{-1}		0.031<br>0.036	[104]
					NOEC (90 d) (n.a)<br>LOEC (90 d) (n.a)	1 ng L^{-1}<br>1 ng L^{-1}		0.1<br>0.1	[105]

Therapeutic group/ Pharmaceutical	Phylum(Class)	Specie (common name)	Acute toxicological endpoint	Acute ecotoxicity data	Chronic toxicological endpoint	Chronic ecotoxicity data	Other remarks	PNEC (ng L^{-1})	Reference
					NOEC (90 d) (n.a)	0.2 ng L^{-1}		0.02	[106]
					LOEC (90 d) (n.a)	2 ng L^{-1}		0.2	
					NOEC (240 d) (FLC, F1, reproduction)	1 ng L^{-1}		0.1	[107]
					LOEC (240 d) (FLC, F1, reproduction)	1 ng L^{-1}		0.1	
					NOEC (42 d) (Sex reversal)	3 ng L^{-1}		0.3	[108]
					LOEC (42 d) (Sex reversal)	3 ng L^{-1}		0.3	
		Gobiocypris rarus			NOEC (240 d) (F1 reproduction)	0.1 ng L^{-1}		0.01	[109]
					VTG NOEC (240 d) (F1 reproduction)	0.2 ng L^{-1}		0.02	
		Pimephales promelas (fathead minnow)			NOEC (29 d) (ELS, growth)	11 ng L^{-1}		1.1	[92]
					NOEC (92 d) (ELS, growth)	1 ng L^{-1}		0.1	
					NOEC (46 d) (PLC	3 ng L^{-1}		0.3	

Therapeutic group/ Pharmaceutical	Phylum(Class)	Specie (common name)	Acute toxicological endpoint	Acute ecotoxicity data	Chronic toxicological endpoint	Chronic ecotoxicity data	Other remarks	PNEC (ng L^{-1})	Reference
					eggs/female, hatch)				
					NOEC (21 d) (fertility)	3 ng L^{-1}		0.3	
					NOEC (21 d) (fertility)	1 ng L^{-1}		0.1	[101]
					LOEC (21 d) (plasma VTG induction)	1 ng L^{-1}		0.1	[110]
					LOEC (21 d) (ultrastructure testes)	1 ng L^{-1}		0.1	
					LOEC (21 d) (ultrastructure liver)	1 ng L^{-1}		0.1	
					LOEC (21 d) (fertilization rate)	10 ng L^{-1}		1	
					NOEC (150 d) (Reproduction)	0.16 ng L^{-1}		0.016	[111]
					NOEC (305 d) (FLC, 2 gen reproduction)	1 ng L^{-1}		0.1	[112]
					LOEC (240 d) (FLC, 2 gen reproduction)	4 ng L^{-1}		0.4	
		Poecilia reticulate (Guppy)			NOEC (108 d) (Reproduction, sex ratio)	44 ng L^{-1}		4.4	[113]

Therapeutic group/ Pharmaceutical	Phylum(Class)	Specie (common name)	Acute toxicological endpoint	Acute ecotoxicity data	Chronic toxicological endpoint	Chronic ecotoxicity data	Other remarks	PNEC (ng L^{-1})	Reference
		Oncorhynchus Mykiss (rainbow trout)			NOEC (60 d) (Reproduction)	8 ng L^{-1}		0.8	[114]
					LOEC (60 d) (Reproduction)	16 ng L^{-1}		1.6	
		Oryzias latipes (Japanese rice fish)			NOEC (100 d) (Intersex)	10 ng L^{-1}		1	[77]
					NOEC (60 d) (Reproduction)	1 ng L^{-1}		0.1	[115]
					LOEC (60 d) (Reproduction)	10 ng L^{-1}		1	
					NOEC (120-180 d) (Reproduction)	2 ng L^{-1}		0.2	[116]
		Rutilis rutilis			NOEC (720 d) (Sex reversal)	0.3 ng L^{-1}		0.03	[117]
					LOEC (720 d) (Sex reversal)	4 ng L^{-1}		0.4	
**Mixtures**									
Algae									
Ibuprofen, fluoxetine and ciprofloxacin	NA	Phytoplankton			LOEC (35 d) (increased in abundance and decreased in diversity)	ibuprofen 600 µg L^{-1},fluoxetine 1,000 µg L^{-1} and ciprofloxacina 1,000 µg L^{-1}	(LOEC was not calculated in the paper)		[118]
Ciprofloxacin and norfloxacin	Chlorophyta (Chlorophyceae)	*Raphidocelis subcapitata (Selenastrum capricornutum,*	EC 50 (72 h) (growth inhibition)	Synergistic effect					[5]

Therapeutic group/ Pharmaceutical	Phylum(Class)	Specie (common name)	Acute toxicological endpoint	Acute ecotoxicity data	Chronic toxicological endpoint	Chronic ecotoxicity data	Other remarks	PNEC (ng L^{-1})	Reference
		Pseudokirchneriella subcapitata)							
Fish									
Ibuprofen , fluoxetine and ciprofloxacin	NA	Zooplankton			LOEC (35 d) (increased abundance and decreases in diversity)	ibuprofen 600 µg L^{-1},fluoxetine 1,000 µg L^{-1} and ciprofloxacina 1,000 µg L^{-1}	(LOEC was not calculated in the paper)		[118]
Ibuprofen, fluoxetine and ciprofloxacin	Chordata (Actinopterygii)	*Lepomis gibbosus*			LOEC (35 d) (mortality)	ibuprofen 60 µg L^{-1},fluoxetine 100 µg L^{-1} and ciprofloxacina 100 µg L^{-1}	(LOEC was not calculated in the paper)		[118]
Fluoxetine, Fluvoxamine, and Sertraline		Cyanobacteria			EC10 (abundance) (7 d)<br>EC50 (abundance) (7 d)<br>EC10 (abundance) (35 d)<br>EC50 (abundance) (35 d)	53.3 nM<br>1040.1 nM<br>198.8 nM<br>957.9 nM			[34]
Fluoxetine, Fluvoxamine, and Sertraline	NA	Chlorophyta			EC10 (abundance) (7 d)	49.4 nM<br>536.6 nM<br>274.9 nM			[34]

Therapeutic group/ Pharmaceutical	Phylum(Class)	Specie (common name)	Acute toxicological endpoint	Acute ecotoxicity data	Chronic toxicological endpoint	Chronic ecotoxicity data	Other remarks	PNEC (ng L^{-1})	Reference
					EC50 (abundance) (7 d)<br>EC10 (abundance) (35 d)<br>EC50 (abundance) (35 d)	1169.4 nM			
Fluoxetine, Fluvoxamine, and Sertraline	NA	Heterokonts			EC10 (abundance) (7 d)<br>EC50 (abundance) (7 d)<br>EC10 (abundance) (35 d)<br>EC50 (abundance) (35 d)	51.4 nM<br>3614.18 nM<br>316.6 nM<br>1346.3 nM			[34]
Fluoxetine, Fluvoxamine, and Sertraline	NA	Cryptophtya/Dino phtya			EC10 (abundance) (7 d)<br>EC50 (abundance) (7 d)<br>EC10 (abundance) (35 d)	15.2 nM<br>99.6 nM<br>152.2 nM<br>664.4 nM			[34]

Therapeutic group/ Pharmaceutical	Phylum(Class)	Specie (common name)	Acute toxicological endpoint	Acute ecotoxicity data	Chronic toxicological endpoint	Chronic ecotoxicity data	Other remarks	PNEC (ng L^{-1})	Reference
					EC50 (abundance) (35 d)				
Sertraline, and Fluoxetine	Arthropoda, Crustacea (Branchiopoda)	*Ceriodaphnia dubia* (Water flea)	LC50 (48 h)	1.09 µM					[119]
Sertraline, and Paroxetine	Arthropoda, Crustacea (Branchiopoda)	*Ceriodaphnia dubia* (Water flea)	LC50 (48 h)	1.71 µM					[119]
Sertraline, and Citalopram	Arthropoda, Crustacea (Branchiopoda)	*Ceriodaphnia dubia* (Water flea)	LC50 (48 h)	5.40 µM					[119]
Fluoxetine, and Paroxetine	Arthropoda, Crustacea (Branchiopoda)	*Ceriodaphnia dubia* (Water flea)	LC50 (48 h)	2.05 µM					[119]
Fluoxetine, and Citalopram	Arthropoda, Crustacea (Branchiopoda)	*Ceriodaphnia dubia* (Water flea)	LC50 (48 h)	6.41 µM					[119]
Paroxetine, and Citalopram	Arthropoda, Crustacea (Branchiopoda)	*Ceriodaphnia dubia* (Water flea)	LC50 (48 h)	8.76 µM					[119]
Fluoxetine, Citalopram, Sertraline and Paroxetine	Arthropoda, Crustacea (Branchiopoda)	*Ceriodaphnia dubia* (Water flea)	LC50 (48 h)	5.48 µM					[119]
Fluoxetine and Triclosan	Chlorophyta (Chlorophyceae)	*Dunaliella tertiolecta*	EC50 (96 h) (population cell density)	135.28 µg L^{-1}					[20]
Sertraline and Diphenhydramine	Arthropoda, Crustacea (Branchiopoda)	*Ceriodaphnia dubia* (Water flea)	LC50 (48 h)	0.433 mg L^{-1}	EC50 (7 d) (reproduction)	0.184 mg L^{-1}			[120]

d - days
EC – Effective concentration
LC – Lethal concentration
LOEC – Lowest effect concentration
NA – Not available
NOEC – No observed effect concentration

n.a – Not available

Table S2. Occurrence data from the different aquatic compartments for environmental risk assessment evaluation [121].

Wastewater influents (WWI) concentrations (ng L^{-1})			
**Therapeutic group**	**Pharmaceutical**	**Median**	**Maximum**
Anx	ALP	0	403.10
	LOR	0	51.30
	ZOL	0	4.33
Antib	AZI	169	578.00
	CLA	324	1891.40
	CIP	196	2706.40
	ERY	92.7	2413.90
Lip Reg	BEZ	270.5	2364.00
	GEM	216	4472.20
	SIM	27	405.70
Antiepi	CAR	193	1832.80
SSRIs	CIT	83	155.10
	N-CIT	143.5	275.90
	ESC	n.a.	32228.00
	FLU	2	571.50
	Nor-FLU	13.7	69.10
	PAR	0	2747.90
	SER	4.1	25.60
	Nor-SER	30.5	72.90
Anti-inf	DIC	449.5	6397.20
	IBU	2680	54963.20
	NAP	1550	29353.10
	PARA	20601	131911.30
Horm	E1	53.9	439.50
	E2	20	596.10
	α-E2	3.7	5008.60
	EE2	4.2	48.20

Wastewater effluents (WWE) concentrations (ng L^{-1})			
**Therapeutic group**	**Pharmaceutical**	**Median**	**Maximum**
Anx	ALP	4.95	16.2
	LOR	61	121
	ZOL	1.5	21.3
Antib	AZI	155	1379.3
	CLA	200	596.7
	CIP	101	501292.40
	ERY	80	1124.5
Lip Reg	BEZ	111.5	948.9
	GEM	181.5	2554.5
	SIM	1	280.1
Antiepi	CAR	226	2016.3
SSRIs	CIT	73	35979.6
	N-CIT	106.5	305.3
	ESC	2.1	688.1
	FLU	5.8	25.7
	Nor-FLU	0	212.9
	PAR	4.5	19.2
	SER	9.35	30.7
	Nor-SER	73	35979.6
Anti-inf	DIC	233	2006
	IBU	163.2	5691.8
	NAP	142	2660.9
	PARA	10	2246.3
Horm	E1	14.4	43.8
	E2	1.35	18.7
	α-E2	0.4	2351.6
	EE2	0.5	2.9

Surface waters (SW) concentrations (ng L^{-1})			
**Therapeutic group**	**Pharmaceutical**	**Median**	**Maximum**
Anx	ALP	1.25	2.2
	LOR	2	22.4
	ZOL	0	0.0
Antib	AZI	n.a.	236.0
	CLA	18.3	183.7
	CIP	20.15	54519.5
	ERY	30	4378.3
Lip Reg	BEZ	21.7	1698.6
	GEM	18.55	1443.0
	SIM	0	8.2
Antiepi	CAR	27.65	680.0
SSRIs	CIT	2.75	15252.8
	N-CIT	n.a.	n.a.
	ESC	n.a.	n.a.
	FLU	0.85	37.9
	Nor-FLU	0	1.4
	PAR	0	3.9
	SER	1.6	102.1
	Nor-SER	2.3	4.5
Anti-inf	DIC	33.5	1928.5
	IBU	0	19.9
	NAP	26	3265.3
	PARA	33.5	2572.6
Horm	E1	2.1	22.9
	E2	1.05	999.8
	α-E2	0	18.5
	EE2	0	64.4

Other water bodies (seawater - SeaW, groundwater - GW and drinking water - DW) concentrations (ng L^{-1})				
**Therapeutic group**	**Pharmaceutical**	**Type of water**	**Median**	**Maximum**
Anx	ALP	DW	2.4	2.4
	LOR	GW	5.1	5.1
Antib	AZI	SeaW	0.14	0.14
		GW	83	83
	CLA	SeaW	0.19	0.19
		GW	5.2	5.2
		DW	2	2
	CIP	SeaW	51.5	51.5
		GW	38.9	38.9
	ERY	SeaW	2.6	2.6
		GW	4.82	4.82
		DW	0.3	0.3
Lip reg	BEZ	GW	2.2	2.2
		MinW	1	1
		DW	27	27
		SeaW	258	258
		GW	35.025	35.025
		MinW	8	8
		DW	0.5	0.5
Antiepi	CAR	SeaW	678	678
		GW	23.3	23.3
		DW	6.4	6.4
SSRIs	CIT	GW	1400	1400
	FLU	GW	10.82	10.82
		DW	0.68	0.68
	Nor-FLU	DW	0.77	0.77
	PAR	GW	1.9	1.9
Anti-inf	DIC	SeaW	200.5	200.5
		GW	75.3	75.3
		MinW	25	25
		DW	16.9	16.9
	4-OH-DIC	GW	15.3	15.3
	IBU	GW	43.585	43.585
		MinW	12	12
		DW	5	5
	NAP	GW	1.015	1.015
		MinW	25	25
		DW	11	11
	PARA	SeaW	116	116
		GW	20	20

		DW	210	210
Horm	E1	GW	2.2	2.2
		DW	0.16	0.16
	E2	GW	0.89	0.89
	α-E2	GW	1	1
	EE2	GW	60.905	60.905

DW – drinking water; GW groundwater; SeaW – seawater; MinW – mineral water; Anx - anxiolytics; Antib - antibiotics; Lip reg - lipid regulators; Antiepi - antiepileptics; SSRIs - Selective serotonin reuptake inhibitors; Anti-inf - anti-inflammatories; Horm – hormones; n.a. – not available.

References:

[1] Y. Li, L. Zhang, X. Liu, J. Ding, Ranking and prioritizing pharmaceuticals in the aquatic environment of China, Sci. Total Environ. 658 (2019) 333–342. doi:10.1016/j.scitotenv.2018.12.048.

[2] J. Vestel, D.J. Caldwell, L. Constantine, V.J. D'Aco, T. Davidson, D.G. Dolan, S.P. Millard, R. Murray-Smith, N.J. Parke, J.J. Ryan, J.O. Straub, P. Wilson, Use of acute and chronic ecotoxicity data in environmental risk assessment of pharmaceuticals, Environ. Toxicol. Chem. 35 (2016) 1201–1212. doi:10.1002/etc.3260.

[3] H. Sidhu, G. O'Connor, D. McAvoy, Risk assessment of biosolids-borne ciprofloxacin and azithromycin, Sci. Total Environ. 651 (2019) 3151–3160. doi:10.1016/j.scitotenv.2018.10.194.

[4] M. Isidori, M. Lavorgna, A. Nardelli, L. Pascarella, A. Parrella, Toxic and genotoxic evaluation of six antibiotics on non-target organisms., Sci. Total Environ. 346 (2005) 87–98. doi:10.1016/j.scitotenv.2004.11.017.

[5] L.-H. Yang, G.-G. Ying, H.-C. Su, J.L. Stauber, M.S. Adams, M.T. Binet, Growth-inhibiting effects of 12 antibacterial agenst and their mixtures on the freshwater microalga Pseudokirchneriella subcapitata., Environ. Toxicol. Chem. 27 (2008) 1201. doi:10.1897/07-471.1.

[6] B. Halling-Sorensen, Environmental risk assessment of antibiotics: comparison of mecillinam, trimethoprim and ciprofloxacin., J. Antimicrob. Chemother. 46 (2000) 53–58. doi:10.1093/jac/46.suppl_1.53.

[7] N. Martins, R. Pereira, N. Abrantes, J. Pereira, F. Gonçalves, C.R. Marques, Ecotoxicological effects of ciprofloxacin on freshwater species: data integration and derivation of toxicity thresholds for risk assessment., Ecotoxicology. 21 (2012) 1167–76. doi:10.1007/s10646-012-0871-x.

[8] I. Ebert, J. Bachmann, U. Kühnen, A. Küster, C. Kussatz, D. Maletzki, C. Schlüter, Toxicity of the fluoroquinolone antibiotics enrofloxacin and ciprofloxacin to photoautotrophic aquatic organisms., Environ. Toxicol. Chem. 30 (2011) 2786–92. doi:10.1002/etc.678.

[9] A.A. Robinson, J.B. Belden, M.J. Lydy, Toxicity of fluoroquinolone antibiotics to aquatic organisms., Environ. Toxicol. Chem. 24 (2005)

423. doi:10.1897/04-210R.1.

[10] X. Nie, X. Wang, J. Chen, V. Zitko, T. An, Response of the freshwater Alga chlorella vulgaris to trichloroisocyanuric acid and ciprofloxacin., Environ. Toxicol. Chem. 27 (2008) 168–73. doi:10.1897/07-028.1.

[11] R.A. Brain, D.J. Johnson, S.M. Richards, H. Sanderson, P.K. Sibley, K.R. Solomon, Effects of 25 pharmaceutical compounds to Lemna gibba using a seven-day static-renewel test., Environ. Toxicol. Chem. 23 (2004) 371–382. doi:10.1897/02-576.

[12] M.D. Machado, E. V. Soares, Sensitivity of freshwater and marine green algae to three compounds of emerging concern, J. Appl. Phycol. 31 (2019) 399–408. doi:10.1007/s10811-018-1511-5.

[13] B. Quinn, F. Gagné, C. Blaise, An investigation into the acute and chronic toxicity of eleven pharmaceuticals (and their solvents) found in wastewater effluent on the cnidarian, Hydra attenuata., Sci. Total Environ. 389 (2008) 306–14. doi:10.1016/j.scitotenv.2007.08.038.

[14] M. Isidori, A. Nardelli, L. Pascarella, M. Rubino, A. Parrella, Toxic and genotoxic impact of fibrates and their photoproducts on non-target organisms., Environ. Int. 33 (2007) 635–41. doi:10.1016/j.envint.2007.01.006.

[15] G.H. Han, H.G. Hur, S.D. Kim, Ecotoxicological risk of pharmaceuticals from wastewater treatment plants in Korea: occurrence and toxicity to Daphnia magna., Environ. Toxicol. Chem. 25 (2006) 265–71. doi:10.1897/05-193R.1.

[16] J.L. Zurita, G. Repetto, A. Jos, M. Salguero, M. López-Artíguez, A.M. Cameán, Toxicological effects of the lipid regulator gemfibrozil in four aquatic systems., Aquat. Toxicol. 81 (2007) 106–15. doi:10.1016/j.aquatox.2006.11.007.

[17] M. Farré, I. Ferrer, A. Ginebreda, M. Figueras, L. Olivella, L. Tirapu, M. Vilanova, D. Barceló, Determination of drugs in surface water and wastewater samples by liquid chromatography–mass spectrometry: methods and preliminary results including toxicity studies with Vibrio fischeri., J. Chromatogr. A. 938 (2001) 187–197. doi:10.1016/S0021-9673(01)01154-2.

[18] C. Mimeault, V.L. Trudeau, T.W. Moon, Waterborne gemfibrozil challenges the hepatic antioxidant defense system and down-regulates peroxisome proliferator-activated receptor beta (PPARbeta) mRNA

levels in male goldfish (Carassius auratus)., Toxicology. 228 (2006) 140–50. doi:10.1016/j.tox.2006.08.025.

[19] D. Raldúa, M. André, P.J. Babin, Clofibrate and gemfibrozil induce an embryonic malabsorption syndrome in zebrafish., Toxicol. Appl. Pharmacol. 228 (2008) 301–14. doi:10.1016/j.taap.2007.11.016.

[20] M.E. DeLorenzo, J. Fleming, Individual and mixture effects of selected pharmaceuticals and personal care products on the marine phytoplankton species Dunaliella tertiolecta., Arch. Environ. Contam. Toxicol. 54 (2008) 203–210. doi:10.1007/s00244-007-9032-2.

[21] P.B. Key, J. Hoguet, L.A. Reed, K.W. Chung, M.H. Fulton, Effects of the statin antihyperlipidemic agent simvastatin on grass shrimp, Palaemonetes pugio., Environ. Toxicol. 23 (2008) 153–60. doi:10.1002/tox.20318.

[22] U. Dahl, E. Gorokhova, M. Breitholtz, Application of growth-related sublethal endpoints in ecotoxicological assessments using a harpacticoid copepod., Aquat. Toxicol. 77 (2006) 433–8. doi:10.1016/j.aquatox.2006.01.014.

[23] B. Ferrari, R. Mons, B. Vollat, B. Fraysse, N. Paxéus, R. Lo Giudice, A. Pollio, J. Garric, Environmental risk assessment of six human pharmaceuticals: are the current environmental risk assessment procedures sufficient for the protection of the aquatic environment?, Environ. Toxicol. Chem. 23 (2004) 1344–54. doi:10.1897/03-246.

[24] B. Ferrari, N. Paxéus, R. Lo Giudice, A. Pollio, J. Garric, Ecotoxicological impact of pharmaceuticals found in treated wastewaters: study of carbamazepine, clofibric acid, and diclofenac., Ecotoxicol. Environ. Saf. 55 (2003) 359–370. doi:10.1016/S0147-6513(02)00082-9.

[25] M. Cleuvers, Aquatic ecotoxicity of pharmaceuticals including the assessment of combination effects., Toxicol. Lett. 142 (2003) 185–194. doi:10.1016/S0378-4274(03)00068-7.

[26] H.J. De Lange, W. Noordoven, A.J. Murk, M. Lürling, E.T.H.M. Peeters, Behavioural responses of Gammarus pulex (Crustacea, Amphipoda) to low concentrations of pharmaceuticals., Aquat. Toxicol. 78 (2006) 209–16. doi:10.1016/j.aquatox.2006.03.002.

[27] J.-W. Kim, H. Ishibashi, R. Yamauchi, N. Ichikawa, Y. Takao, M. Hirano, M. Koga, K. Arizono, Acute toxicity of pharmaceutical and personal care products on freshwater crustacean (Thamnocephalus

platyurus) and fish (Oryzias latipes)., J. Toxicol. Sci. 34 (2009) 227–232. doi:10.2131/jts.34.227.

[28] Y. Kim, K. Choi, J. Jung, S. Park, P.-G. Kim, J. Park, Aquatic toxicity of acetaminophen, carbamazepine, cimetidine, diltiazem and six major sulfonamides, and their potential ecological risks in Korea., Environ. Int. 33 (2007) 370–5. doi:10.1016/j.envint.2006.11.017.

[29] A.M. Christensen, S. Faaborg-Andersen, F. Ingerslev, A. Baun, Mixture and single-substance toxicity of selective serotonin reuptake inhibitors toward algae and crustaceans., Environ. Toxicol. Chem. 26 (2007) 85. doi:10.1897/06-219R.1.

[30] T.B. Henry, J.W. Kwon, K.L. Armbrust, M.C. Black, Acute and chronic toxicity of five selective serotonin reuptake inhibitors in Ceriodaphnia dubia, Environ. Toxicol. Chem. 23 (2004) 2229–2233. doi:10.1897/03-278.

[31] P.P. Fong, N. Molnar, Antidepressants cause foot detachment from substrate in five species of marine snail., Mar. Environ. Res. 84 (2013) 24–30. doi:10.1016/j.marenvres.2012.11.004.

[32] B.W. Brooks, P.K. Turner, J.K. Stanley, J.J. Weston, E. a Glidewell, C.M. Foran, M. Slattery, T.W. La Point, D.B. Huggett, Waterborne and sediment toxicity of fluoxetine to select organisms., Chemosphere. 52 (2003) 135–142. doi:10.1016/S0045-6535(03)00103-6.

[33] B.W. Brooks, C.M. Foran, S.M. Richards, J. Weston, P.K. Turner, J.K. Stanley, K.R. Solomon, M. Slattery, T.W. La Point, Aquatic ecotoxicology of fluoxetine., Toxicol. Lett. 142 (2003) 169–183. doi:10.1016/S0378-4274(03)00066-3.

[34] D.J. Johnson, H. Sanderson, R.A. Brain, C.J. Wilson, K.R. Solomon, Toxicity and hazard of selective serotonin reuptake inhibitor antidepressants fluoxetine, fluvoxamine, and sertraline to algae., Ecotoxicol. Environ. Saf. 67 (2007) 128–139. doi:10.1016/j.ecoenv.2006.03.016.

[35] J. Neuwoehner, K. Fenner, B.I. Escher, Physiological modes of action of fluoxetine and its human metabolites in algae., Environ. Sci. Technol. 43 (2009) 6830–6837. doi:10.1021/es9005493.

[36] J.K. Stanley, A.J. Ramirez, C.K. Chambliss, B.W. Brooks, Enantiospecific sublethal effects of the antidepressant fluoxetine to a model aquatic vertebrate and invertebrate., Chemosphere. 69 (2007)

9–16. doi:10.1016/j.chemosphere.2007.04.080.

[37] A.R.R. Péry, M. Gust, B. Vollat, R. Mons, M. Ramil, G. Fink, T. Ternes, J. Garric, Fluoxetine effects assessment on the life cycle of aquatic invertebrates., Chemosphere. 73 (2008) 300–304. doi:10.1016/j.chemosphere.2008.06.029.

[38] G. Nałecz-Jawecki, Evaluation of the in vitro biotransformation of fluoxetine with HPLC, mass spectrometry and ecotoxicological tests., Chemosphere. 70 (2007) 29–35. doi:10.1016/j.chemosphere.2007.07.035.

[39] G. Nentwig, Effects of pharmaceuticals on aquatic invertebrates. Part II: the antidepressant drug fluoxetine., Arch. Environ. Contam. Toxicol. 52 (2007) 163–170. doi:10.1007/s00244-005-7190-7.

[40] M. Gust, T. Buronfosse, L. Giamberini, M. Ramil, R. Mons, J. Garric, Effects of fluoxetine on the reproduction of two prosobranch mollusks: Potamopyrgus antipodarum and Valvata piscinalis., Environ. Pollut. 157 (2009) 423–429. doi:10.1016/j.envpol.2008.09.040.

[41] P.D. Hazelton, W.G. Cope, S. Mosher, T.J. Pandolfo, J.B. Belden, M.C. Barnhart, R.B. Bringolf, Fluoxetine alters adult freshwater mussel behavior and larval metamorphosis., Sci. Total Environ. 445–446 (2013) 94–100. doi:10.1016/j.scitotenv.2012.12.026.

[42] D. Caminada, C. Escher, K. Fent, Cytotoxicity of pharmaceuticals found in aquatic systems: comparison of PLHC-1 and RTG-2 fish cell lines., Aquat. Toxicol. 79 (2006) 114–123. doi:10.1016/j.aquatox.2006.05.010.

[43] T.B. Henry, M.C. Black, Acute and chronic toxicity of fluoxetine (selective serotonin reuptake inhibitor) in western mosquitofish., Arch. Environ. Contam. Toxicol. 54 (2008) 325–330. doi:10.1007/s00244-007-9018-0.

[44] Y. Nakamura, H. Yamamoto, J. Sekizawa, T. Kondo, N. Hirai, N. Tatarazako, The effects of pH on fluoxetine in Japanese medaka (Oryzias latipes): acute toxicity in fish larvae and bioaccumulation in juvenile fish., Chemosphere. 70 (2008) 865–873. doi:10.1016/j.chemosphere.2007.06.089.

[45] M.M. Schultz, M.M. Painter, S.E. Bartell, A. Logue, E.T. Furlong, S.L. Werner, H.L. Schoenfuss, Selective uptake and biological consequences of environmentally relevant antidepressant

pharmaceutical exposures on male fathead minnows., Aquat. Toxicol. 104 (2011) 38–47. doi:10.1016/j.aquatox.2011.03.011.

[46] S.M. Richards, S.E. Cole, A toxicity and hazard assessment of fourteen pharmaceuticals to Xenopus laevis larvae., Ecotoxicology. 15 (2006) 647–656. doi:10.1007/s10646-006-0102-4.

[47] V.L. Cunningham, D.J.C. Constable, R.E. Hannah, Environmental Risk Assessment of Paroxetine., Environ. Sci. Technol. 38 (2004) 3351–3359. doi:10.1021/es035119x.

[48] E. Minagh, R. Hernan, K. O'Rourke, F.M. Lyng, M. Davoren, Aquatic ecotoxicity of the selective serotonin reuptake inhibitor sertraline hydrochloride in a battery of freshwater test species., Ecotoxicol. Environ. Saf. 72 (2009) 434–440. doi:10.1016/j.ecoenv.2008.05.002.

[49] K. Lamichhane, S.N. Garcia, D.B. Huggett, D.L. DeAngelis, T.W. La Point, Exposures to a selective serotonin reuptake inhibitor (SSRI), sertraline hydrochloride, over multiple generations: Changes in life history traits in Ceriodaphnia dubia., Ecotoxicol. Environ. Saf. 101 (2014) 124–130. doi:10.1016/j.ecoenv.2013.11.026.

[50] T.W. Valenti, P.P. Hurtado, C.K. Chambliss, B.W. Brooks, Aquatic toxicity of sertraline to Pimephales promelas at environmentally relevant surface water pH., Environ. Toxicol. Chem. 28 (2009) 2685–2694.

[51] J.R. Lawrence, G.D.W. Swerhone, E. Topp, D.R. Korber, T.R. Neu, L.I. Wassenaar, Structural and functional responses of river biofilm communities to the nonsteroidal anti-inflammatory diflofenac., Environ. Toxicol. Chem. 26 (2007) 573. doi:10.1897/06-340R.1.

[52] M. Cleuvers, Chronic Mixture Toxicity of Pharmaceuticals to Daphnia – The Example of Nonsteroidal Anti-Inflammatory Drugs., in: K. Kümmerer (Ed.), Pharm. Environ., 2006: pp. 277–284.

[53] T. Haap, R. Triebskorn, H.-R. Köhler, Acute effects of diclofenac and DMSO to Daphnia magna: immobilisation and hsp70-induction., Chemosphere. 73 (2008) 353–9. doi:10.1016/j.chemosphere.2008.05.062.

[54] G. Nalecz-Jawecki, G. Persoone, Toxicity of selected pharmaceuticals to the Anostracan crustacean Thamnocephalus platyurus - Comparison of sublethal and lethal effect levels with the 1h Rapidtoxkit and the 24h Thamnotoxkit microbiotests., Environ.

Sci. Pollut. Res. - Int. 13 (2006) 22–27. doi:10.1065/espr2006.01.005.

[55] U. Memmert, A. Peither, R. Burri, K. Weber, T. Schmidt, J.P. Sumpter, A. Hartmann, Diclofenac: New data on chronic toxicity and bioconcentration in fish., Environ. Toxicol. Chem. 32 (2013) 442–52. doi:10.1002/etc.2085.

[56] J. Schwaiger, H. Ferling, U. Mallow, H. Wintermayr, R.D. Negele, Toxic effects of the non-steroidal anti-inflammatory drug diclofenac. Part I: histopathological alterations and bioaccumulation in rainbow trout., Aquat. Toxicol. 68 (2004) 141–50. doi:10.1016/j.aquatox.2004.03.014.

[57] R. Triebskorn, H. Casper, a Heyd, R. Eikemper, H.-R. Köhler, J. Schwaiger, Toxic effects of the non-steroidal anti-inflammatory drug diclofenac. Part II: cytological effects in liver, kidney, gills and intestine of rainbow trout (Oncorhynchus mykiss)., Aquat. Toxicol. 68 (2004) 151–66. doi:10.1016/j.aquatox.2004.03.015.

[58] A.C. Mehinto, E.M. Hill, C.R. Tyler, Uptake and biological effects of environmentally relevant concentrations of the nonsteroidal anti-inflammatory pharmaceutical diclofenac in rainbow trout (Oncorhynchus mykiss)., Environ. Sci. Technol. 44 (2010) 2176–82. doi:10.1021/es903702m.

[59] B. Hoeger, B. Köllner, D.R. Dietrich, B. Hitzfeld, Water-borne diclofenac affects kidney and gill integrity and selected immune parameters in brown trout (Salmo trutta f. fario)., Aquat. Toxicol. 75 (2005) 53–64. doi:10.1016/j.aquatox.2005.07.006.

[60] J.L. Oaks, M. Gilbert, M.Z. Virani, R.T. Watson, C.U. Meteyer, B.A. Rideout, H.L. Shivaprasad, S. Ahmed, M.J. Iqbal Chaudhry, M. Arshad, S. Mahmood, A. Ali, A. Ahmed Khan, Diclofenac residues as the cause of vulture population decline in Pakistan., Nature. 427 (2004) 630–633. doi:10.1038/nature02317.

[61] S. Ortiz de García, G.P. Pinto, P.A. García-Encina, R.I. Mata, Ranking of concern, based on environmental indexes, for pharmaceutical and personal care products: An application to the Spanish case, J. Environ. Manage. 129 (2013) 384–397. doi:10.1016/j.jenvman.2013.06.035.

[62] F. Pomati, A.G. Netting, D. Calamari, B.A. Neilan, Effects of erythromycin, tetracycline and ibuprofen on the growth of Synechocystis sp. and Lemna minor., Aquat. Toxicol. 67 (2004) 387–96. doi:10.1016/j.aquatox.2004.02.001.

[63] L.-H. Heckmann, A. Callaghan, H.L. Hooper, R. Connon, T.H. Hutchinson, S.J. Maund, R.M. Sibly, Chronic toxicity of ibuprofen to Daphnia magna: Effects on life history traits and population dynamics., Toxicol. Lett. 172 (2007) 137–45. doi:10.1016/j.toxlet.2007.06.001.

[64] G. Dave, G. Herger, Determination of detoxification to Daphnia magna of four pharmaceuticals and seven surfactants by activated sludge., Chemosphere. 88 (2012) 459–66. doi:10.1016/j.chemosphere.2012.02.070.

[65] N. Pounds, S. Maclean, M. Webley, D. Pascoe, T. Hutchinson, Acute and chronic effects of ibuprofen in the mollusc Planorbis carinatus (Gastropoda: Planorbidae)., Ecotoxicol. Environ. Saf. 70 (2008) 47–52. doi:10.1016/j.ecoenv.2007.07.003.

[66] X. Yang, X. Xu, X. Wei, J. Wan, Y. Zhang, Biomarker effects in carassius auratus exposure to ofloxacin, sulfamethoxazole and ibuprofen, Int. J. Environ. Res. Public Health. 16 (2019). doi:10.3390/ijerph16091628.

[67] M. Cleuvers, Mixture toxicity of the anti-inflammatory drugs diclofenac, ibuprofen, naproxen, and acetylsalicylic acid., Ecotoxicol. Environ. Saf. 59 (2004) 309–15. doi:10.1016/S0147-6513(03)00141-6.

[68] M. Isidori, M. Lavorgna, A. Nardelli, A. Parrella, L. Previtera, M. Rubino, Ecotoxicity of naproxen and its phototransformation products., Sci. Total Environ. 348 (2005) 93–101. doi:10.1016/j.scitotenv.2004.12.068.

[69] J.T. Sherer, Pharmaceuticals in the environment., Am. J. Heal. Pharm. 63 (2006) 174–178. doi:10.2146/ajhp050123.

[70] R. El-Bassat, H. Touliabah, G. Harisa, Toxicity of four pharmaceuticals from different classes to isolated plankton species., African J. Aquat. Sci. 37 (2012) 71–80. doi:10.2989/16085914.2012.666376.

[71] M.-H. Li, Acute toxicity of 30 pharmaceutically active compounds to freshwater planarians, Dugesia japonica., Toxicol. Environ. Chem. 95 (2013) 1157–1170. doi:10.1080/02772248.2013.857671.

[72] Q. Li, P. Wang, L. Chen, H. Gao, L. Wu, Acute toxicity and histopathological effects of naproxen in zebrafish (Danio rerio) early life stages., Environ. Sci. Pollut. Res. 23 (2016) 18832–18841.

doi:10.1007/s11356-016-7092-4.

[73] K.-P. Henschel, A. Wenzel, M. Diedrich, A. Fliedner, Environmental Hazard Assessment of Pharmaceuticals., Regul. Toxicol. Pharmacol. 25 (1997) 220–225. doi:10.1006/rtph.1997.1102.

[74] R. Kühn, M. Pattard, K. Pernak, A. Winter, Results of the harmful effects of selected water pollutants (anilines, phenols, aliphatic compounds) to Daphnia magna., Water Res. 23 (1989) 495–499. doi:10.1016/0043-1354(89)90141-3.

[75] L.W. Sun, M.M. Qu, Y.Q. Li, Y.L. Wu, Y.G. Chen, Z.M. Kong, Z.T. Liu, Toxic effects of aminophenols on aquatic life using the Zebrafish embryo test and the comet assay., Bull. Environ. Contam. Toxicol. 73 (2004) 628–634. doi:10.1007/s00128-004-0474-1.

[76] M. Li, Acute toxicity of industrial endocrine-disrupting chemicals, natural and synthetic sex hormones to the freshwater planarian, Dugesia japonica., Toxicol. Environ. Chem. 95 (2013) 984–991. doi:10.1080/02772248.2013.840376.

[77] C.D. Metcalfe, T.L. Metcalfe, Y. Kiparissis, B.G. Koenig, C. Khan, R.J. Hughes, T.R. Croley, R.E. March, T. Potter, Estrogenic potency of chemicals detected in sewage treatment plant effluents as determined by in vivo assays with Japanese medaka (Oryzias latipes)., Environ. Toxicol. Chem. 20 (2001) 297–308. doi:10.1002/etc.5620200210.

[78] S. Imai, J. Koyama, K. Fujii, Effects of estrone on full life cycle of Java medaka (Oryzias javanicus), a new marine test fish., Environ. Toxicol. Chem. 26 (2007) 726–31. doi:10.1897/05-539R2.1.

[79] K.L. Thorpe, R. Benstead, T.H. Hutchinson, C.R. Tyler, Associations between altered vitellogenin concentrations and adverse health effects in fathead minnow (Pimephales promelas)., Aquat. Toxicol. 85 (2007) 176–183. doi:10.1016/j.aquatox.2007.08.012.

[80] M. Seki, S. Fujishima, T. Nozaka, M. Maeda, K. Kobayashi, Comparison of response to 17β-estradiol and 17β-trenbolone among three small fish species., Environ. Toxicol. Chem. 25 (2006) 2742. doi:10.1897/05-647R.1.

[81] F. Brion, C.. Tyler, X. Palazzi, B. Laillet, J.. Porcher, J. Garric, P. Flammarion, Impacts of 17β-estradiol, including environmentally relevant concentrations, on reproduction after exposure during embryo-larval-, juvenile- and adult-life stages in zebrafish (Danio

rerio)., Aquat. Toxicol. 68 (2004) 193–217. doi:10.1016/j.aquatox.2004.01.022.

[82] L.T.M. Van der Ven, E.-J. Van den Brandhof, J.H. Vos, P.W. Wester, Effects of the estrogen agonist 17β-estradiol and antagonist tamoxifen in a partial life-cycle assay with with zebrafish (danio rerio)., Environ. Toxicol. Chem. 26 (2007) 92. doi:10.1897/06-092R1.1.

[83] J.P. Nash, D.E. Kime, L.T.M. Van der Ven, P.W. Wester, F. Brion, G. Maack, P. Stahlschmidt-Allner, C.R. Tyler, Long-term exposure to environmental concentrations of the pharmaceutical ethynylestradiol causes reproductive failure in fish., Environ. Health Perspect. 112 (2004) 1725–1733. doi:10.1289/ehp.7209.

[84] G.M. Cripe, B.L. Hemmer, L.R. Goodman, J.W. Fournie, S. Raimondo, J.C. Vennari, R.L. Danner, K. Smith, B.R. Manfredonia, D.H. Kulaw, M.J. Hemmer, Multigerational exposure of the estuarine Sheepshead minnow (cyprinodon variegatus) to 17β-estradiol. I. Organism-level effects over three generations., Environ. Toxicol. Chem. 28 (2009) 2397. doi:10.1897/08-542.1.

[85] T. Liao, Q.L. Guo, S.W. Jin, W. Cheng, Y. Xu, Comparative responses in rare minnow exposed to 17β-estradiol during different life stages., Fish Physiol. Biochem. 35 (2009) 341–349. doi:10.1007/s10695-008-9247-9.

[86] C.A. Pollino, E. Georgiades, D.A. Holdway, Use of the Australian crimson-spotted rainbowfish (Melanotaenia fluviatilis) as a model test species for investigating the effects of endocrine disruptors., Environ. Toxicol. Chem. 26 (2007) 2171. doi:10.1897/06-603R.1.

[87] I.J. Kang, H. Yokota, Y. Oshima, Y. Tsuruda, T. Yamaguchi, M. Maeda, N. Imada, H. Tadokoro, T. Honjo, Effect of 17β-estradiol on the reproduction of Japanese medaka (Oryzias latipes)., Chemosphere. 47 (2002) 71–80. doi:10.1016/S0045-6535(01)00205-3.

[88] N. Hirai, A. Nanba, M. Koshio, T. Kondo, M. Morita, N. Tatarazako, Feminization of Japanese medaka (Oryzias latipes) exposed to 17β-estradiol: Formation of testis–ova and sex-transformation during early-ontogeny., Aquat. Toxicol. 77 (2006) 78–86. doi:10.1016/j.aquatox.2005.11.001.

[89] M. Seki, H. Yokota, M. Maeda, K. Kobayashi, Fish full life-cycle testing for 17beta-estradiol on medaka (Oryzias latipes)., Environ. Toxicol. Chem. 24 (2005) 1259–66. doi:10.1897/03-26.

[90] J.A. Jukosky, M.C. Watzin, J.C. Leiter, The effects of environmentally relevant mixtures of estrogens on Japanese medaka (Oryzias latipes) reproduction., Aquat. Toxicol. 86 (2008) 323–331. doi:10.1016/j.aquatox.2007.11.012.

[91] S. Imai, J. Koyama, K. Fujii, Effects of 17β-estradiol on the reproduction of Java-medaka (Oryzias javanicus), a new test fish species., Mar. Pollut. Bull. 51 (2005) 708–714. doi:10.1016/j.marpolbul.2005.02.018.

[92] D.J. Caldwell, F. Mastrocco, P.D. Anderson, R. Länge, J.P. Sumpter, Predicted-no-effect concentrations for the steroid estrogens estrone, 17β-estradiol, estriol, and 17α-ethinylestradiol., Environ. Toxicol. Chem. 31 (2012) 1396–1406. doi:10.1002/etc.1825.

[93] V.J. Kramer, S. Miles-Richardson, S.L. Pierens, J.P. Giesy, Reproductive impairment and induction of alkaline-labile phosphate, a biomarker of estrogen exposure, in fathead minnows (Pimephales promelas) exposed to waterborne 17β-estradiol., Aquat. Toxicol. 40 (1998) 335–360. doi:10.1016/S0166-445X(97)00060-X.

[94] N.W. Shappell, K.H. Elder, M. West, Estrogenicity and nutrient concentration of surface waters surrounding a large confinement dairy operation using best management practices for land application of animal wastes., Environ. Sci. Technol. 44 (2010) 2365–2371. doi:10.1021/es903669m.

[95] G. Toft, E. Baatrup, Altered sexual characteristics in guppies (Poecilia reticulata) exposed to 17β-estradiol and 4-tert-octylphenol during sexual development., Ecotoxicol. Environ. Saf. 56 (2003) 228–237. doi:10.1016/S0147-6513(02)00138-0.

[96] C.D. Robinson, E. Brown, J.A. Craft, I.M. Davies, C. Megginson, C. Miller, C.F. Moffat, Bioindicators and reproductive effects of prolonged 17β-oestradiol exposure in a marine fish, the sand goby (Pomatoschistus minutus)., Aquat. Toxicol. 81 (2007) 397–408. doi:10.1016/j.aquatox.2006.12.020.

[97] C.O. Onogbosele, Bioavailability of organic contaminants in rivers., Brunel University London, 2015. http://bura.brunel.ac.uk/handle/2438/11050.

[98] W. Jaser, Effects of 17α-ethinylestradiol on the reproduction of the cladoceran species Ceriodaphnia reticulata and Sida crystallina., Environ. Int. 28 (2003) 633–638. doi:10.1016/S0160-4120(02)00101-0.

[99] D.J. Caldwell, F. Mastrocco, T.H. Hutchinson, R. Länge, D. Heijerick, C. Janssen, P.D. Anderson, J.P. Sumpter, Derivation of an Aquatic Predicted No-Effect Concentration for the Synthetic Hormone, 17α-Ethinyl Estradiol., Environ. Sci. Technol. 42 (2008) 7046–7054. doi:10.1021/es800633q.

[100] G.F. Vandenbergh, D. Adriaens, T. Verslycke, C.R. Janssen, Effects of 17α-ethinylestradiol on sexual development of the amphipod Hyalella azteca., Ecotoxicol. Environ. Saf. 54 (2003) 216–222. doi:10.1016/S0147-6513(02)00030-1.

[101] S. Jobling, D. Casey, T. Rodgers-Gray, J. Oehlmann, U. Schulte-Oehlmann, S. Pawlowski, T. Baunbeck, A.. Turner, C.. Tyler, Comparative responses of molluscs and fish to environmental estrogens and an estrogenic effluent., Aquat. Toxicol. 66 (2004) 207–222. doi:10.1016/j.aquatox.2004.01.002.

[102] E.J. Zillioux, I.C. Johnson, Y. Kiparissis, C.D. Metcalfe, J. V. Wheat, S.G. Ward, H. Liu, The sheepshead minnow as an in vivo model for endocrine disruption in marine teleosts: A partial life-cycle test with 17α-ethynylestradiol., Environ. Toxicol. Chem. 20 (2001) 1968–1978. doi:10.1002/etc.5620200915.

[103] S. Örn, H. Holbech, T.H. Madsen, L. Norrgren, G.I. Petersen, Gonad development and vitellogenin production in zebrafish (Danio rerio) exposed to ethinylestradiol and methyltestosterone., Aquat. Toxicol. 65 (2003) 397–411. doi:10.1016/S0166-445X(03)00177-2.

[104] C. Schäfers, M. Teigeler, A. Wenzel, G. Maack, M. Fenske, H. Segner, Concentration- and time-dependent effects of the synthetic estrogen, 17α-ethinylestradiol, on reproductive capabilities of the Zebrafish, Danio rerio., J. Toxicol. Environ. Heal. Part A. 70 (2007) 768–779. doi:10.1080/15287390701236470.

[105] K. Van den Belt, P. Berckmans, C. Vangenechten, R. Verheyen, H. Witters, Comparative study on the in vitro/in vivo estrogenic potencies of 17β-estradiol, estrone, 17α-ethynylestradiol and nonylphenol., Aquat. Toxicol. 66 (2004) 183–195. doi:10.1016/j.aquatox.2003.09.004.

[106] H. Xu, J. Yang, Y. Wang, Q. Jiang, H. Chen, H. Song, Exposure to 17α-ethynylestradiol impairs reproductive functions of both male and female zebrafish (Danio rerio)., Aquat. Toxicol. 88 (2008) 1–8. doi:10.1016/j.aquatox.2008.01.020.

[107] J. Soares, A.M. Coimbra, M.A. Reis-Henriques, N.M. Monteiro,

M.N. Vieira, J.M.A. Oliveira, P. Guedes-Dias, A. Fontaínhas-Fernandes, S.S. Parra, A.P. Carvalho, Disruption of zebrafish (Danio rerio) embryonic development after full life-cycle parental exposure to low levels of ethinylestradiol., Aquat. Toxicol. 95 (2009) 330–338. doi:10.1016/j.aquatox.2009.07.021.

[108] M. Fenske, G. Maack, C. Schäfers, H. Segner, An environmentally relevant concentration of estrogen induces arrest of male gonad development in zebrafish, Danio rerio., Environ. Toxicol. Chem. 24 (2005) 1088–1098. doi:10.1897/04-096R1.1.

[109] J. Zha, L. Sun, Y. Zhou, P.A. Spear, M. Ma, Z. Wang, Assessment of 17α-ethinylestradiol effects and underlying mechanisms in a continuous, multigeneration exposure of the Chinese rare minnow (Gobiocypris rarus)., Toxicol. Appl. Pharmacol. 226 (2008) 298–308. doi:10.1016/j.taap.2007.10.006.

[110] S. Pawlowski, R. van Aerle, C.. Tyler, T. Braunbeck, Effects of 17α-ethinylestradiol in a fathead minnow (Pimephales promelas) gonadal recrudescence assay., Ecotoxicol. Environ. Saf. 57 (2004) 330–345. doi:10.1016/j.ecoenv.2003.07.019.

[111] J.L. Parrott, B.R. Blunt, Life-cycle exposure of fathead minnows (Pimephales promelas) to an ethinylestradiol concentration below 1 ng/L reduces egg fertilization success and demasculinizes males., Environ. Toxicol. 20 (2005) 131–141. doi:10.1002/tox.20087.

[112] R. Länge, T.H. Hutchinson, C.P. Croudace, F. Siegmund, H. Schweinfurth, P. Hampe, G.H. Panter, J.P. Sumpter, Effects of the synthetic estrogen 17 alpha-ethinylestradiol on the life-cycle of the fathead minnow (Pimephales promelas)., Environ. Toxicol. Chem. 20 (2001) 1216–1227. doi:10.1002/etc.5620200610.

[113] T. Kristensen, E. Baatrup, M. Bayley, 17α-Ethinylestradiol Reduces the Competitive Reproductive Fitness of the Male Guppy (Poecilia reticulata)1, Biol. Reprod. 72 (2005) 150–156. doi:10.1095/biolreprod.104.033001.

[114] I.R. Schultz, A. Skillman, J.-M. Nicolas, D.G. Cyr, J.J. Nagler, Short-term exposure to 17α-ethynylestradiol decreases the fertility of sexually maturing male rainbow trout (Oncorhynchus mykiss)., Environ. Toxicol. Chem. 22 (2003) 1272–1280. doi:10.1002/etc.5620220613.

[115] S. Scholz, 17-α-ethinylestradiol affects reproduction, sexual differentiation and aromatase gene expression of the medaka (Oryzias

latipes), Aquat. Toxicol. 50 (2000) 363–373. doi:10.1016/S0166-445X(00)00090-4.

[116] G.C. Balch, C.A. Mackenzie, C.D. Metcalfe, Alterations of gonadal development and reproductive success in Japanese medaka (Oryzias latipes) exposed to 17α-ethinylestradiol., Environ. Toxicol. Chem. 23 (2004) 782. doi:10.1897/02-539.

[117] A. Lange, Y. Katsu, R. Ichikawa, G.C. Paull, L.L. Chidgey, T.S. Coe, T. Iguchi, C.R. Tyler, Altered sexual development in roach (Rutilus rutilus) exposed to environmental concentrations of the pharmaceutical 17α-Ethinylestradiol and associated expression dynamics of aromatases and estrogen receptors, Toxicol. Sci. 106 (2008) 113–123. doi:10.1093/toxsci/kfn151.

[118] S.M. Richards, C.J. Wilson, D.J. Johnson, D.M. Castle, M. Lam, S.A. Mabury, P.K. Sibley, K.R. Solomon, Effects of pharmaceutical mixtures in aquatic microcosms., Environ. Toxicol. Chem. 23 (2004) 1035–42. doi:10.1897/02-616.

[119] T.B. Henry, M.C. Black, Mixture and single-substance acute toxicity of selective serotonin reuptake inhibitors in Ceriodaphnia dubia., Environ. Toxicol. Chem. 26 (2007) 1751–5. doi:10.1897/06-265R.1.

[120] E.W. Goolsby, C.M. Mason, J.T. Wojcik, A.M. Jordan, M.C. Black, Acute and chronic effects of diphenhydramine and sertraline mixtures in Ceriodaphnia dubia., Environ. Toxicol. Chem. 32 (2013) 2866–9. doi:10.1002/etc.2378.

[121] A. Pereira, L. Silva, C. Laranjeiro, C. Lino, A. Pena, Selected Pharmaceuticals in Different Aquatic Compartments: Part I—Source, Fate and Occurrence, Molecules. 25 (2020) 1026. doi:10.3390/molecules25051026.
